# Oxidation Strength of PLA Filled with Algal Biomass and Rosemary Extract Powders for Food-Safe Handling

**DOI:** 10.3390/polym18040504

**Published:** 2026-02-18

**Authors:** Traian Zaharescu, Marius Bumbac, Cristina Mihaela Nicolescu, Aurora Craciun, Radu Mirea

**Affiliations:** 1 Radiochemistry Center, National Institute for R&D in Electrical Engineering ICPE-CA Bucharest, 030138 Bucharest, Romania; traian.zaharescu@icpe-ca.ro; 2 Faculty of Science and Arts, Valahia University of Targoviste, 130004 Targoviste, Romania; 3 Institute of Multidisciplinary Research for Science and Technology, Valahia University of Targoviste, 130004 Targoviste, Romania; 4 Faculty of Medicine, University Ovidius Constanta, 900527 Constanta, Romania; aurora.craciun@365.univ-ovidius.ro; 5 Romanian Research and Development Institute for Gas Turbines—COMOTI, 061125 Bucharest, Romania; radu.mirea@comoti.ro

**Keywords:** poly(lactic acid), *Ascophyllum nodosum*, *Arthrospira platensis*, rosemary extract, multifunctional fillers

## Abstract

Poly(lactic acid) (PLA) is widely used in food-contact applications due to its bio-based origin, compostability, and transparency; however, its limited resistance to thermo-oxidative degradation remains a challenge for applications involving repeated thermal exposure. The moderate but repetitive heating conditions commonly encountered during food use and pre-recycling stages were analyzed for the samples filled with algal biomass and rosemary extract, additives accepted for use in the food industry. In this context, the present study introduces a comparative and application-driven approach by evaluating the effect of food-grade fillers—rosemary extract, spirulina biomass, and kelp biomass—incorporated at low loadings (0.5–3 wt%) on the thermal and oxidative behavior of PLA subjected to repeated heating at 80 °C. The presented results show algal biomasses as multifunctional fillers and benchmark their performance against a well-established natural extract. By combining DSC, FTIR, and chemiluminescence analyses, the study aims to clarify whether such bio-fillers act as stabilizing or destabilizing factors under realistic service-life thermal stress. This strategy provides insight into the suitability of algae-based fillers for food-contact PLA materials from both performance and recyclability perspectives.

## 1. Introduction

Poly(lactic acid) (PLA) is one of the most widely used bio-based and biodegradable polymers, particularly in the food packaging sector, due to its renewability, compostability, and good transparency [1]. However, despite these advantages, PLA suffers from several inherent drawbacks that strongly limit its application in high-performance food contact materials. These drawbacks include low thermal stability, poor resistance to thermo-oxidative degradation, brittleness, and limited barrier properties against oxygen and moisture. Furthermore, PLA is prone to degradation during melt processing, leading to chain scission, molecular weight reduction, and deterioration of mechanical properties [2,3,4]. To overcome these limitations, the incorporation of functional fillers has emerged as a promising strategy [5,6]. In this context, *Rosmarinus officinalis* (rosemary, RM) extract, *Arthrospira platensis* (spirulina, SP) dry biomass, and *Ascophyllum nodosum* (kelp, K) dry biomass were embedded into a PLA matrix (same commercial sort) at three different concentrations (0.5, 1, and 3 wt%). These additives are all accepted in the food industry, ensuring compatibility with food contact regulations while providing added functional value [7,8,9,10].

The incorporation of natural biofillers such as rosemary extract and algae biomass, including spirulina and kelp (*Ascophyllum nodosum*), into polylactic acid (PLA) is increasingly recognized as an effective approach to developing multifunctional, food-contact–compliant biocomposites that enhance both material performance during use and biodegradation at the end of life [10]. Rosemary extract, already widely used in the food industry as a natural antioxidant, is rich in phenolic diterpenes such as carnosic acid and carnosol, which act as efficient radical scavengers and metal chelators. When incorporated into PLA, it functions as a stabilizing additive during melt processing and service life, limiting thermo-oxidative degradation and polymer chain scission, thereby improving thermal stability, color retention, mechanical ductility, and durability [11,12,13,14,15,16]. Spirulina, a protein- and polysaccharide-rich microalgal biomass containing phycocyanin, carotenoids, and phenolic compounds, offers complementary multifunctionality by providing antioxidant and antimicrobial activity alongside particulate reinforcement, while benefiting from its established approval as a food ingredient [17,18,19,20,21]. Similarly, kelp biomass contains alginates, fucoidans, polyphenols, and minerals capable of interacting with the PLA matrix, contributing to increased stiffness, improved barrier properties, and additional antioxidant protection, while remaining a low-cost, renewable, and food-grade filler [13,22,23]. Collectively, these natural additives mitigate molecular weight degradation during processing, enhance gas barrier performance through the creation of tortuous diffusion pathways, and, in the case of algae biomass, significantly accelerate PLA biodegradation by promoting hydrolytic and fungal attack via hydrophilic domains that facilitate microbial adhesion and polymer fragmentation [24,25]. Although algae-filled PLA systems may exhibit moderate reductions in tensile strength and changes in glass transition temperature, their improved degradation kinetics, environmental compatibility, and economic competitiveness make them attractive candidates for sustainable food packaging and related applications, while advances in interfacial adhesion further extend their potential into automotive and biomedical fields such as tissue engineering, wound dressing, and drug delivery [26,27]. The present study shows a comparison between a conventional natural antioxidant (rosemary extract) and emerging algal biomass fillers (spirulina and kelp). While rosemary extract is already known for its strong antioxidant efficiency, the present strategy investigates whether whole microalgal and macroalgal biomasses can provide comparable stabilization effects, combined with mechanical and barrier enhancements. This comparison enables the evaluation of algae-based fillers as sustainable, multifunctional alternatives to conventional plant extracts. The incorporation of *Rosmarinus officinalis* extract, *Arthrospira platensis*, and *Ascophyllum nodosum* into PLA at different loadings is a scientifically justified and industrially relevant strategy to overcome the intrinsic limitations of PLA. By using fillers already accepted in the food industry, this approach ensures regulatory compatibility while introducing antioxidant, reinforcing, and barrier-enhancing functionalities. The comparative assessment of rosemary extract versus algal biomasses further contributes to the development of next-generation, fully bio-based and functional packaging materials.

## 2. Materials and Methods

### 2.1. Materials

High-molecular-weight poly(lactic acid) pellets, Ingeo™ Biopolymer 2003D, were purchased from Nature Works LLC (Minnetonka, MN, USA) with a density of 1.24 g∙cm^−3^ and a melt flow index of 6 g/10 min (210 °C, 2.16 kg). The dry rosemary biomass and the algal biomass in powder form with 5.8 ± 0.5% (*w*/*w*) moisture content were purchased from Hyperici Pharm SRL, Târgoviște, Romania. All reagents used in the experiment were of analytical grade and were purchased from Fisher Scientific (Waltham, MA, USA).

The additive powders: *Rosmarinus officinalis* (RM), *Arthrospira platensis*—spirulina (SP), and *Ascophyllum nodosum* (K) were embedded in three different concentrations (0.5, 1, and 3 wt%) by their solubilization into the mother solution of PLA in chloroform. Soxhlet extraction was carried out using ethanol under reflux conditions, which exposed the extract to temperatures close to the boiling point of ethanol (~78 °C) throughout the extraction process. After filtration, the solid fraction was dried at 80 °C for 4 h to ensure complete removal of residual solvent. This drying temperature is comparable to the thermal conditions experienced during extraction and was therefore not expected to introduce additional degradation. Rosemary extract contains thermally stable polyphenolic compounds, such as carnosic acid and rosmarinic acid, which retain antioxidant activity after moderate thermal exposure. In addition, the drying temperature was selected to reflect the intended application of the extract as a filler in PLA-based food packaging materials designed for contact with hot food, where exposure to similar temperatures is anticipated. Accordingly, the applied thermal treatment is representative of practical processing and use conditions.

The notation of the samples, as correlated with the experimental procedure, is presented in Table S1 (Supplementary Material).

### 2.2. Sample Preparation

The PLA samples were dissolved in chloroform to obtain homogeneous solutions with a concentration of 10% (*w*/*v*). This concentration was selected to ensure sufficient fluidity while avoiding the formation of highly viscous mixtures, thereby enabling good dispersion of the fillers in the PLA matrix. The fillers were subsequently added in amounts corresponding to 0.5, 1, and 3 wt% relative to the dry PLA content in the chloroform solution, and the mixtures were thoroughly dispersed. Each resulting solution was cast into a round Petri dish to form thin films, and the solvent was allowed to evaporate at room temperature. After complete drying, the films were cut into small chips, which were used as test specimens for subsequent measurements.

### 2.3. Thermal Ageing

Thermal ageing of PLA specimens was carried out in an electrical drying oven (model 15-103-0508, Fisher Scientific, Campus Drive, Mundelein, IL, USA) at 80 °C for three heating cycles of 8 h each. The ageing temperature of 80 °C was selected because PLA is commonly used in food industry applications at temperatures below this threshold. Above 60–70 °C, PLA begins to soften and lose mechanical strength, making 80 °C a relevant condition for evaluating thermal stability.

### 2.4. Chemiluminescence

The evaluation of thermal stability in correlation with the antioxidative efficiency was accurately accomplished by both chemiluminescence procedures [28,29]: isothermal determinations, when the progress of oxidation is monitored at constant temperature, and nonisothermal measurements, when the collection of expelled photons is visualized over the whole temperature range. Chemiluminescence (CL) measurements were conducted using a Lumipol 3 spectrometer (Polymer Institute, Slovak Academy of Sciences, Bratislava, Slovakia), with measurement uncertainties of ±0.3 °C (temperature) and ±50 Hz s^−1^ (photon counting). The polymer samples were weighed (up to 20 mg) and placed in 20 µL aluminum trays. The selected values of heating rate used in our CL measurements are 5, 10, 15, and 20 °C min^−1^, while the value of temperature applied in the isothermal CL investigation is 170 °C. In isothermal chemiluminescence (CL) studies of PLA, a temperature of 170 °C was selected because it provides an optimal balance between experimental sensitivity, practical relevance, and controlled degradation kinetics. This temperature lies above the melting range of PLA (approximately 140–155 °C, according to the technical datasheet), ensuring that the polymer is fully in the molten state, with sufficient molecular mobility and homogeneous oxygen diffusion to allow oxidative processes to develop uniformly throughout the material. At the same time, 170 °C is high enough for thermo-oxidative degradation to progress at a convenient and measurable rate within reasonable experimental times, without triggering excessively rapid or uncontrolled thermal decomposition. Importantly, this temperature is also commonly used in extrusion and melt-processing operations for PLA, making the isothermal CL conditions directly relevant to real processing environments. Consequently, CL measurements performed at 170 °C enable reliable monitoring of hydroperoxide formation and radical reactions under melt-state conditions representative of industrial processing, providing meaningful insight into the oxidative stability and stabilization efficiency of PLA-based systems.

### 2.5. Fourier Transform Infrared Spectroscopy

The spectral data were recorded on Vertex 80 infrared spectrometer (Bruker, Karlsruhe, Germany) provided with an ATR investigation system. The scanning range was 4000 cm^−1^ to 400 cm^−1^ at a spectral resolution of 4 cm^−1^ and 32 scans.

### 2.6. Differential Scanning Calorimetry (DSC)

Differential scanning calorimetry (DSC) measurements were performed using a DSC 3+ STAR^e^ system (Mettler Toledo, Greifensee, Switzerland). The polymer samples were analyzed over a temperature range of 30 to 250 °C at a heating rate of 10 °C min^−1^. Approximately 10 ± 1 mg of each sample was weighed using a Secura 225D-1CEU analytical balance (Sartorius, Gottingen, Germany) with a precision of 1 × 10^−5^ g and placed in standard 40 µL aluminum pans. An empty 40 µL aluminum pan was used as the reference. Recorded thermograms were then processed with the use of StarE software v. 20.00 (Mettler Toledo GMBH), and thus thermal characteristic parameters were calculated.

## 3. Results

PLA is a versatile biopolymer with a wide range of applications [30], including medical textiles, food packaging, and polymer recycling. It also serves as an excellent substrate for composite production [31]. During oxidative degradation, PLA exhibits a distinctive behavior characterized by two competing radical-driven processes resulting from macromolecular fragmentation: (i) oxidation, similar to that observed in hydrocarbon polymers [32], and (ii) continuous fragmentation through a backbiting mechanism, forming cyclic oligomers [33].

Backbiting in PLA should not be interpreted in the classical sense used for hydrocarbon polymers. Unlike polyolefins, PLA does not contain tertiary carbons, and intramolecular hydrogen abstraction-driven radical backbiting is therefore not the dominant degradation pathway. In PLA, chain scission primarily proceeds through intramolecular transesterification and ester bond cleavage mechanisms, which are promoted by thermal energy and moisture and may be assisted by oxidative processes. Although PLA lacks tertiary carbon centers, radical formation can still occur via hydrogen abstraction from secondary sp^3^ carbon sites located α to ester groups. These sites are relatively susceptible due to stabilization of the resulting macroradicals by adjacent electron-withdrawing carbonyl and ester functionalities. Once initiated, thermo-oxidative reactions can contribute indirectly to backbiting by generating macroradicals and oxygenated species, as well as by promoting the formation of low-molecular-weight oxidation products, including water, within the polymer matrix. At elevated temperatures, the presence of moisture further accelerates ester hydrolysis and intramolecular transesterification, resulting in a coupled degradation mechanism in which radical-assisted oxidation facilitates hydrolytic chain scission rather than acting as the sole driving force.

The interplay between these processes provides insight into the material’s ageing state [34,35]. Incorporating antioxidants into PLA formulations is a key strategy for enhancing durability [36], as these additives improve stabilization against oxidative degradation. However, the performance of PLA modified with specific antioxidants may also reveal the polymer’s inherent tendency toward depolymerization [37]. At elevated temperatures (≈180–250 °C), backbiting becomes the dominant degradation pathway, causing molecular weight reduction, increased melt flow index, formation of volatile cyclic lactide, and loss of mechanical and thermal properties. Furthermore, thermal analysis of additives offers valuable information regarding product quality at different stages of the circular economy [38].

### 3.1. Isothermal CL Measurements

**Figure 1:**
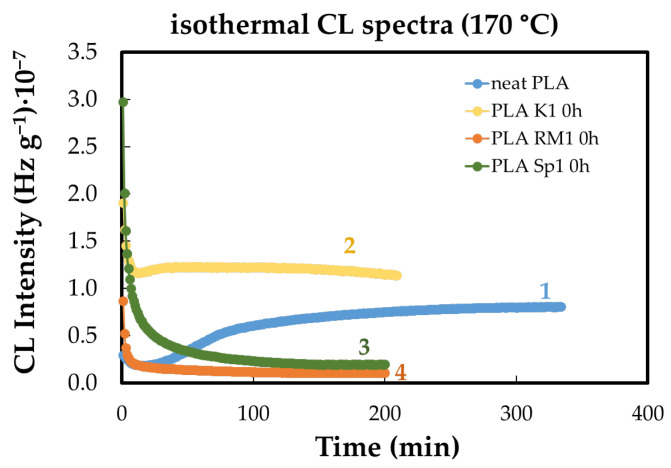
The isothermal CL spectra recorded on the PLA-based samples (heating temperature: 170 °C). Composition: (1) neat PLA; (2) PLA/K; (3) PLA/SP; (4) PLA/RM (additive loading: 1 wt%).

Isothermal CL measurements are used to assess the sensitivity of materials to oxidation at specific temperatures. This susceptibility is quantified by the oxidation induction time (OIT), which indicates the thermal stability of the sample. Additionally, the oxidation rate (*v_ox_*), representing the progression of degradation during the propagation stage, can be determined from the slope of the CL curve. The relative positions of the isothermal CL curves reflect the stability ranking of the samples and the reactions occurring during oxidation propagation. Figure 1 illustrates how sample formulation influences degradation behavior, acting as a structural factor that delays oxidation by hindering the conversion of molecular fragments into hydroperoxide-derived products. The shapes of curves 2–4 suggest that the additive contributes to inhibiting free radical oxidation, whereas the pristine polymer begins to oxidize after approximately 20 min (Figure 1).

In the isothermal chemiluminescence measurements presented in Figure 1, stabilization does not necessarily imply the complete suppression of oxidative reactions, but rather a modification of their kinetics. In the case of PLA/K, the observed sustained CL plateau reflects the establishment of a steady-state regime in which the rates of hydroperoxide formation and decomposition are balanced, resulting in a constant light emission intensity. This behavior differs from the rapid intensity increase observed for the pristine PLA, which indicates an accelerated propagation stage following oxidation onset. The absence of a pronounced intensity rise for PLA/K, together with the lower overall CL level, suggests that the additive limits the accumulation of reactive oxidation products and slows radical propagation. Therefore, the plateau is interpreted as evidence of oxidative stabilization through rate moderation rather than complete inhibition, consistent with a reduced oxidation rate (*v_ox_*) and delayed degradation progression.

The stabilization efficiency of PLA is closely linked to antioxidant activity. Antioxidants interfere with oxidation processes, shortening the time required to reach the steady ageing state and indicating how rapidly the material may degrade.

Chemiluminescence measurements primarily probe radical-mediated thermoxidative processes associated with the formation and decomposition of hydroperoxides, and therefore do not directly evidence polymer chain scission or depolymerization. In the case of PLA, degradation is commonly described as a multistep mechanism in which initial radical oxidation occurs preferentially at secondary C (sp^3^) sites, leading to the formation of oxygenated products, including hydroperoxides and low-molecular-weight species. At elevated temperatures, water generated during oxidative reactions, or present as residual moisture, may subsequently participate in hydrolytic chain scission. Accordingly, depolymerization is considered here as a secondary process associated with advanced stages of thermal degradation, inferred from established PLA degradation pathways rather than directly from CL data.

**Figure 2:**
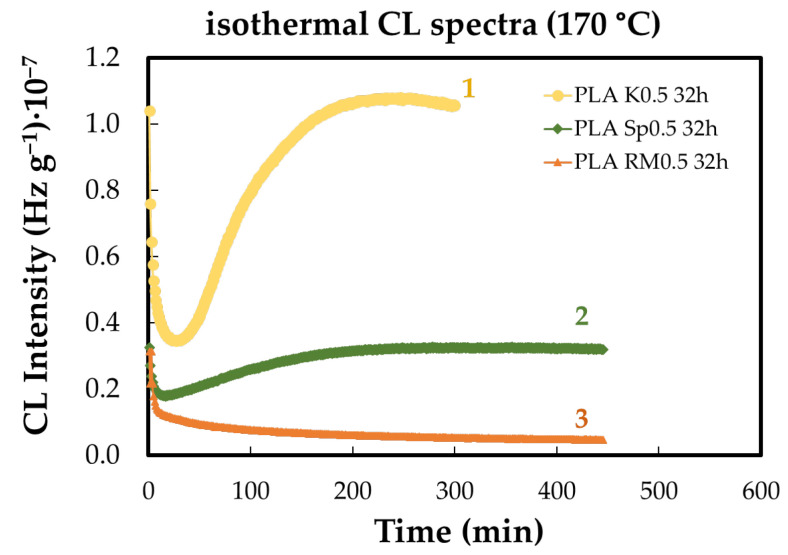
The isothermal CL spectra recorded on the PLA samples modified with natural antioxidants (preheating time: 4 cycles of 8 h each. Heating temperature: 170 °C). Composition: (1) PLA/K; (2) PLA/SP; (3) PLA/RM (additive loading: 0.5 wt%).

In pristine PLA, oxidation and depolymerization remain in competition for more than 100 min, despite an oxidation induction time (OIT) of only 10 min. Polymer degradation alters the balance between free-radical generation and hydroperoxide accumulation, which initiates the oxidation chain. As shown in Figure 2, pre-heating affects PLA modified with natural antioxidants by changing the relative contributions of two key processes: the antioxidative action of the additives and the accumulation and evolution of lactides and polylactides as stable end products.

The essential modifications of material structure by thermal treatment in air lead to an increase in the amount of hydroperoxide that corresponds to the activity of the additive. The comparison of oxidation period for the two states of PLA degradation suggests that the material stabilization is effectively done in the first 50 min of measurements.

The PLA/K curve in Figure 2 shows a moderated and gradually evolving CL profile, in contrast to the sharper intensity increase observed for neat PLA, which is characteristic of rapid oxidation propagation. The presence of the additive therefore alters the balance between radical formation and termination, leading to a slower oxidation rate despite sustained emission. Consequently, the observed CL response is interpreted as kinetic stabilization through controlled oxidation progression rather than increased oxidative activity.

**Figure 3:**
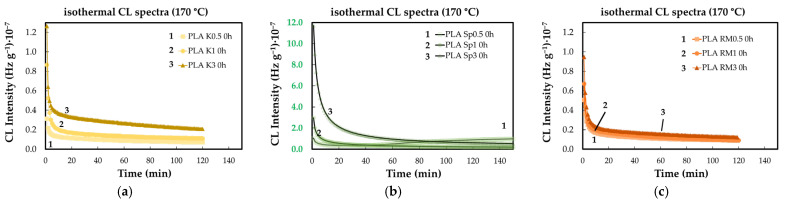
The isothermal CL spectra recorded on unaged PLA stabilized with natural antioxidants (testing temperature: 170 °C). Additive: (**a**) K; (**b**) SP; (**c**) RM (concentrations: (1) 0.5 wt%; (2) 1 wt%; (3) 3 wt%).

The effects of concentration brought about by each of the studied AOs are revealed in Figure 3. The functioning of the additive indicates the opposite contributions that increase the stabilization activities as their loadings enhance [39]. The reason for this discrepancy involves the competition of the two ways of behavior—stabilization and inactivity—with the conversion of radical intermediates into lactides as final degradation products.

As may be observed, there are also some dissimilarities between the progress of degradation in the PLA samples containing various loadings of additives. While the lowest concentration of SP (Figure 3c) exhibits its antioxidant properties, determining a long OIT (41 min), the other measurements display a continuous decrease in the emitted photons during the CL measurements, representing the controlled thermal ageing. The other difference may be noticed between the effects brought about by K and RM. They do not interfere with the chain formation of lactones, because the substitution of an active proton is not possible. The decrease in the oxidation rates of PLA containing K and RM is probably due to the disagreement between the distinct levels of participation.

### 3.2. Nonisothermal CL Measurements

**Figure 4:**
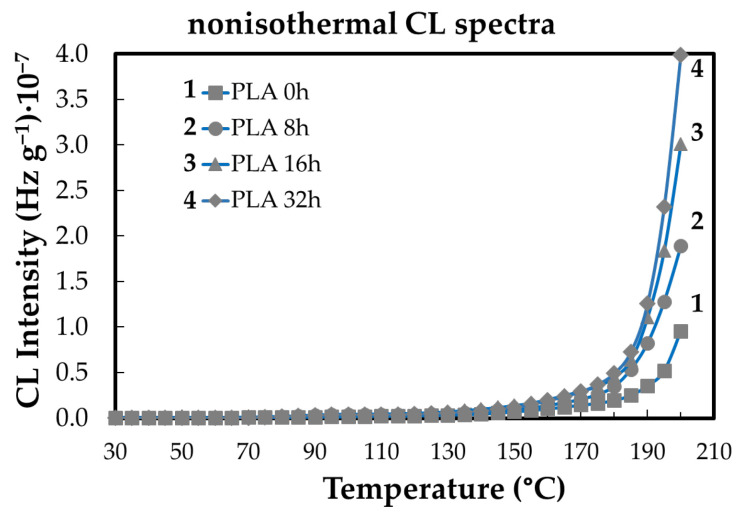
The nonisothermal CL spectra obtained on the neat PLA aged at 80 °C for different heating periods (heating rate: 5 °C min^−1^) (heating time: (1) non; (2) one cycle of 8 h; (3) two cycles of 8 h; (4) four cycles of 8 h).

Non-isothermal CL measurements provide insight into the evolution of PLA degradation as a function of temperature and ageing state (Figure 4). As the temperature increases, PLA undergoes thermally induced chain scission, which generates additional reactive sites and low-molecular-weight fragments, thereby increasing the material’s susceptibility to oxidative reactions. Below approximately 100 °C, oxidative activity remains limited due to insufficient thermal activation of radical formation. At higher temperatures, particularly above 150 °C, the accumulation of oxidation products becomes clearly detectable, leading to a continuous increase in CL intensity. This behavior reflects the temperature-driven kinetics of radical generation and propagation, with ongoing molecular fragmentation continuously supplying oxidizable intermediates throughout the heating process.

**Figure 5:**
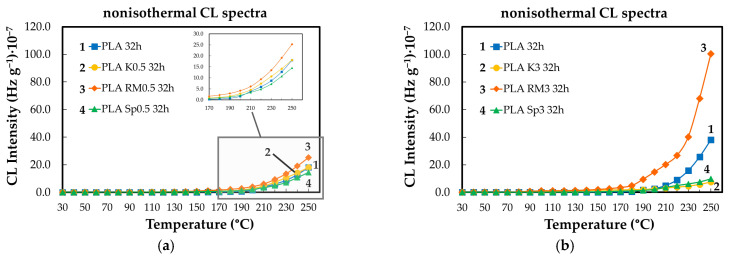
The nonisothermal CL spectra were recorded on the PLA modified with three natural antioxidants after their aging for four cycles of 8 h (heating rate: 10 °C min^−1^). AO concentration: (**a**) 0.5 wt%; (**b**) 3 wt%. PLA modification state: (1) non; (2) K; (3) RM, (4) SP.

The contribution of different additives to the evolution of the oxidation state of PLA specimens depends on the type of this component (Figure 5). In fact, the relative ability shown by the studied stabilizers would prove the importance of the manufacturing procedure when a certain composition shows a peculiar interrelation between the polymer support and the added compounds. The comparison between the two formulations illustrated in Figure 5 reveals the extension of the stability temperature range for the higher amounts of minor component (3 wt%).

**Figure 6:**
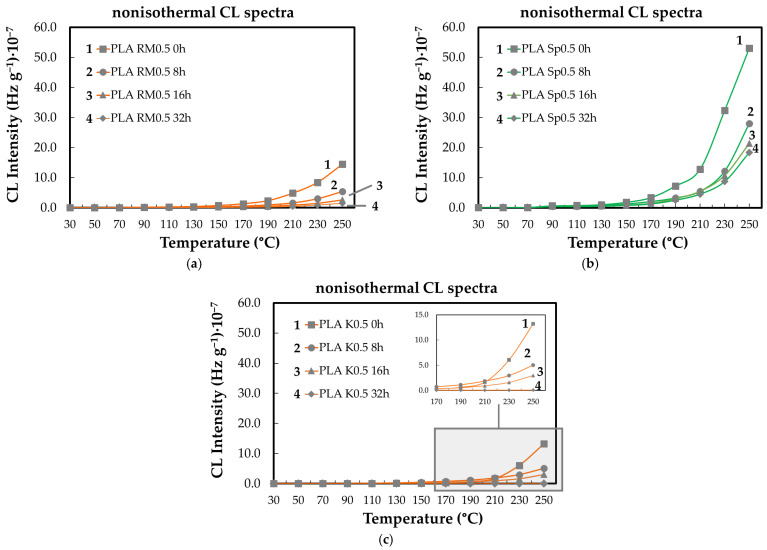
The nonisothermal CL spectra of PLA/stabilizers (0.5 wt%) aged at various times (heating rate: 20 °C min^−1^). Added compounds: (**a**) RM, (**b**) SP, (**c**) K. Heating intervals: (1) none; (2) one cycle of 8 h; (3) two cycles of 8 h; (4) four cycles of 8 h.

Figure 6 illustrates the effect of thermal treatment time at 80 °C on the subsequent oxidation behavior of PLA-based systems. The results indicate that prolonged exposure at this temperature alters the evolution of CL intensity, reflecting changes in the concentration and reactivity of oxidation precursors formed during pre-ageing. Samples subjected to longer treatment times exhibit a more pronounced increase in CL intensity at elevated temperatures, consistent with the accumulation of thermally generated intermediates that promote oxidation during subsequent heating. These observations suggest that the differences observed among the samples arise primarily from the extent of thermal history rather than from a concentration-dependent stabilization mechanism. Accordingly, the CL response is interpreted as a consequence of time-dependent structural and chemical modifications induced during treatment at 80 °C, which influence the kinetics of oxidative degradation.

### 3.3. FTIR Analysis

FTIR analysis was made to identify and quantify chemical and structural changes in PLA induced by moderate thermal treatment and by the presence of fillers (natural antioxidant powders), and to relate these changes to the oxidation and stability trends observed by chemiluminescence (CL) and differential scanning calorimetry (DSC). FTIR is used to track modifications in functional groups that are directly connected to the main degradation pathways of PLA—thermo-oxidation, chain scission, and secondary reactions leading to low-molecular products.

**Figure 7:**
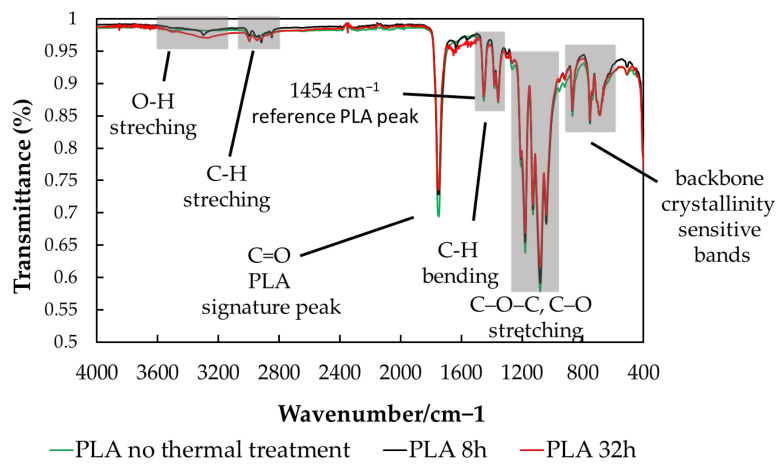
FTIR spectra of PLA: no thermal treatment (green line), 8 h, and 32 h thermal treatment.

Figure 7 shows the IR spectra of PLA samples subjected to mild thermal treatment for 8 and 32 h, compared with untreated PLA. Only these spectra are included to emphasize the bands characteristic of the PLA structure clearly and to avoid overcrowding the figure with overlapping spectra that display identical curve shapes. IR analysis of all samples, regardless of filler presence or thermal treatment, reveals no shifts in the characteristic PLA bands. The spectra in Figure 7 therefore illustrate the typical PLA absorption bands, as summarized in Table 1.

In PLA, intramolecular transesterification (“backbiting”) leading to the formation of cyclic oligomers and lactide has been reported to produce spectral changes in the 920–960 cm^−1^ region, where lactide-related ring vibrations may overlap with PLA skeletal and crystallinity-associated bands. An increase in intensity or the emergence of additional features within this region has therefore been associated with advanced depolymerization processes. In the present study, no new absorption bands or systematic intensity changes were observed between 920 and 960 cm^−1^ following thermal treatment at 80 °C for 8–32 h, either in neat PLA or in filled samples. In addition, the positions of the characteristic PLA absorption bands remained unchanged with thermal treatment and filler addition. These results indicate that intramolecular transesterification and backbiting-type depolymerization do not occur to a detectable extent under the applied mild thermal conditions.

Another IR feature commonly associated with transesterification is the carbonyl stretching region. Although subtle changes in the 1700–1800 cm^−1^ region can occur as crystallinity and morphology evolve during thermal cycling, no such changes are observed here. The shape and position of the carbonyl absorption band remain unchanged for all samples, regardless of thermal treatment or filler addition, indicating the absence of detectable transesterification-related effects.

The band at 1454 cm^−1^, assigned to CH_3_ asymmetric bending/deformation, is commonly used as an internal reference for PLA and lactide because it is present in both species and is relatively insensitive to morphological changes. Accordingly, this band was used for normalization in the absorbance ratios discussed below.

**Figure 8:**
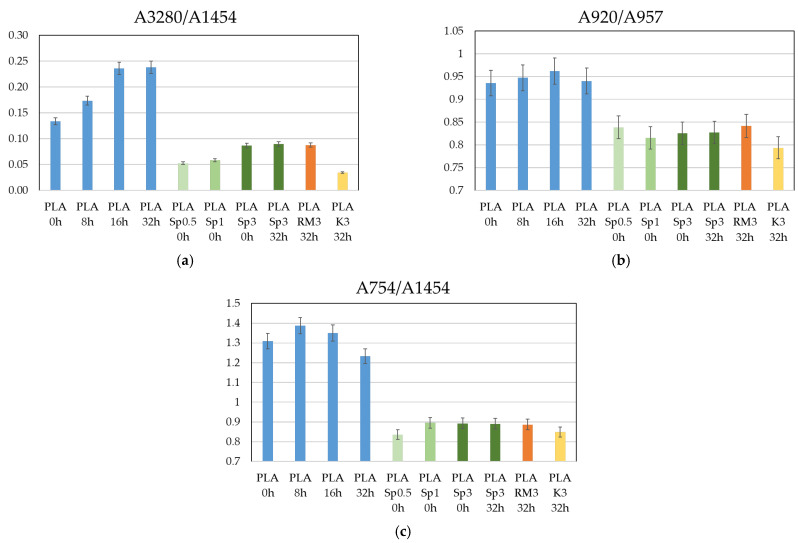
Values of ratios of: (**a**) absorbance measured at 3280 cm^−1^ relative to the absorbance measured at reference wavelength 1454 cm^−1^, (**b**) absorbance measured at 920 cm^−1^ relative to the absorbance measured at reference wavelength 957 cm^−1^, and (**c**) absorbance measured at 754 cm^−1^ relative to the absorbance measured at reference wavelength 1454 cm^−1^.

Figure 8a presents the A3280/A1454 ratio, where the band at ~3280 cm^−1^ is associated with O–H stretching and can be correlated with the formation of hydroxyl end groups resulting from polymer degradation at sp^3^ carbon sites. Neat PLA exhibits higher A3280/A1454 values than the filled samples, and this ratio increases with thermal treatment time, reaching a maximum after 16 h at 80 °C. Prolonging the treatment to 32 h does not lead to a further increase, indicating a saturation in newly formed hydroxyl groups. In contrast to neat PLA, the filled systems (green, orange, and yellow bars) do not display a clear or systematic evolution of the A3280/A1454 ratio with thermal treatment time or filler concentration. The absence of a monotonic trend suggests that, within the investigated conditions, hydroxyl group formation is effectively suppressed and remains close to a steady level. Although the presence of fillers slightly influences the intensity of the 1454 cm^−1^ band, this effect is considered secondary, as the largest decrease in the A3280/A1454 ratio is observed for the lowest spirulina biomass content, where the contribution of the filler to the 1454 cm^−1^ band is expected to be minimal. This supports the conclusion that the reduced ratio primarily reflects a lower extent of O–H formation rather than an artefact arising from changes in the reference band. Considering that thermo-oxidative degradation of neat PLA preferentially initiates at secondary sp^3^ carbon sites adjacent to ester groups, which are particularly susceptible to hydrogen abstraction due to the stabilizing effect of neighboring electron-withdrawing carbonyl functionalities, the consistently lower A3280/A1454 values in the filled samples indicate that the fillers hinder oxidation at these sites. The lack of differentiation among the various fillers further suggests that the stabilizing effect is general in nature, likely related to radical scavenging or oxygen-diffusion-limiting mechanisms, rather than to specific chemical differences between the biomass types.

Figure 8b shows the A920/A957 ratio, used as an indicator of the relative crystalline (≈920 cm^−1^) versus amorphous (≈956–957 cm^−1^) contribution in PLA. Neat PLA displays the highest A920/A957 values, indicating the largest relative crystalline contribution. Thermal treatment up to 16 h increases this ratio, consistent with annealing-induced chain ordering and crystal perfection above Tg. After 32 h, a slight decrease is observed, which can be attributed to saturation of crystallization and the onset of competing effects such as chain scission, hydrolysis, or structural reorganization. All filled samples exhibit lower A920/A957 values than neat PLA, indicating a reduced relative crystalline contribution and/or an increased amorphous or interfacial constrained fraction. The evolution of this ratio with treatment time is not monotonic in the composites, which is typical of filled systems where competing effects—such as limited nucleation, restricted chain mobility near the filler surface, filler dispersion variability, and the formation of a rigid amorphous fraction—act simultaneously.

Figure 8c presents the A754/A1454 ratio to assess morphology-related changes induced by thermal treatment and filler addition. The band at ~754 cm^−1^ lies in the fingerprint region of PLA and is associated with skeletal and backbone-related vibrations that are sensitive to chain conformation, packing, and crystallinity, even in the absence of chemical changes. For neat PLA, A754/A1454 increases slightly after thermal treatment up to 16 h, consistent with annealing-induced ordering, and then decreases after 32 h, suggesting the influence of degradation or hydrolytic effects that limit further crystallization. In contrast, all filled samples show lower and nearly constant A754/A1454 values, independent of filler type, loading, or treatment time. Notably, the evolution of A754/A1454 closely follows the trend observed for A920/A957 (Figure 8b), indicating that both ratios reflect similar morphology-related changes induced by filler incorporation and mild thermal treatment rather than chemical degradation.

### 3.4. DSC Analysis

**Figure 9:**
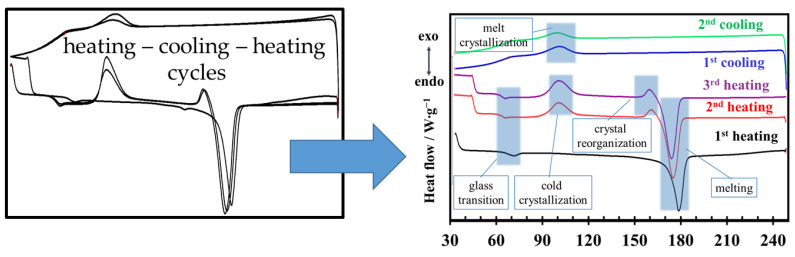
DSC curve pattern of the tested PLA samples.

DSC analysis shows that all PLA samples—whether thermally treated or untreated, and whether filled or unfilled—exhibit the same overall thermal behavior. As illustrated in Figure 9, the DSC thermograms recorded over successive heating–cooling cycles display a common pattern characterized by the glass transition, cold crystallization, melting during heating, and melt crystallization upon cooling. No additional thermal events are detected as a result of thermal treatment or filler incorporation; the observed differences among samples are limited to shifts in peak positions and changes in peak width or intensity within these characteristic regions.

The DSC data presented in Figure 9 show that the glass transition occurs in the temperature range of approximately 55–75 °C. The position and shape of the glass transition differ between the first heating cycle and the subsequent second and third heating cycles. In addition, cold crystallization and pre-melting events are not observed during the first heating step. This initial heating reflects the complex thermal history of the samples, in which residual crystallinity from processing, disordered chain orientation, and non-uniform dispersion of nucleation sites, particularly in filled samples, contribute to a broad and poorly defined crystallization behavior. As a result, crystallization does not initiate uniformly throughout the material, but instead occurs over a wider temperature interval, leading to peak broadening. After the first heating–cooling cycle, a more homogeneous population of nucleation centers is established. Consequently, during the second and third heating cycles, well-defined cold crystallization and pre-melting peaks become apparent, as shown in Figure 9. The pre-melting events refer to endothermic features occurring below the main melting peak of PLA and does not imply a distinct thermodynamic phase transition preceding complete melting. These events are commonly associated with structural rearrangements within the semicrystalline morphology, such as the melting of less-perfect or thinner lamellae, crystal reorganization, or the relaxation of constrained amorphous regions formed during prior thermal history. Accordingly, the term is used here to describe minor endothermic processes related to crystal perfection and reorganization occurring prior to the bulk melting of PLA.

#### 3.4.1. Glass Transition

DSC measurements were performed on PLA samples in air over the temperature range of 30–250 °C using repeated heating–cooling cycles. For neat (unfilled) PLA (Supplementary Material, Figure S1a), the onset temperature of the glass transition during the first heating cycle increases progressively with thermal treatment at 80 °C, from ~62 °C for the untreated sample to ~67 °C after 32 h of treatment. At the same time, the heat capacity change at Tg (ΔCp) decreases after 8 h of thermal exposure and remains nearly constant for longer treatment times. The simultaneous increase in Tg, onset and decrease in ΔCp indicate a reduction in the fraction of mobile amorphous chains and the development of a more constrained and uniformly organized amorphous phase induced by mild thermal annealing.

In contrast, during the second and third heating DSC cycles, all neat PLA samples exhibit similar Tg, onset values around ~58 °C, demonstrating that the initial differences originate from the prior thermal history and are removed after the first heating–cooling cycle. The partial recovery of ΔCp observed in the third heating cycle compared with the second suggests some relaxation and redistribution of chain constraints upon repeated thermal cycling. Overall, these results indicate that mild thermal treatment at 80 °C primarily affects the amorphous-phase organization of PLA rather than inducing permanent chemical or structural degradation.

Although the thermal history is erased after the first DSC heating–cooling cycle, thermally treated PLA samples continue to exhibit lower ΔCp values in the second and third heating steps compared with untreated PLA. This indicates that mild annealing at 80 °C induces persistent constraints in the amorphous phase, reducing the fraction of mobile amorphous chains. In contrast, untreated PLA retains a larger mobile amorphous fraction after thermal cycling, resulting in higher ΔCp values. These results suggest that the thermal treatment leads to a more constrained and structurally stabilized amorphous phase rather than reversible thermal-history effects.

For the filled PLA samples, the evolution of the glass-transition onset temperature and ΔCp generally follows the same trends observed for neat PLA (Figure S1b–m). Thermal treatment at 80 °C leads to lower Tg, onset values and higher ΔCp in the subsequent DSC heating cycles, indicating partial relaxation of the initial thermal history.

In PLA filled with kelp biomass (Figure S1b–e), some deviations are observed. For the untreated composite, ΔCp decreases, while Tg, onset increases from ~65–66 °C in the first heating cycle to ~66–68 °C in the second and third cycles. For samples thermally treated for 8 and 16 h, Tg, onset increases from ~58–60 °C in the first heating to ~64–68 °C in the subsequent heating cycles. In the case of the 32 h–treated samples, ΔCp shows a similar evolution, while Tg, onset depends on kelp content, being lower for samples without kelp or with 1 wt% kelp and higher by approximately 2–4 °C for samples containing 0.5 wt% or 3 wt% kelp.

PLA samples containing rosemary extract (Figure S1f–i) show trends in Tg, onset and ΔCp similar to those of neat PLA. However, for all thermally treated samples, Tg, onset is consistently higher than in unfilled PLA, independent of treatment time and DSC heating cycle. This behavior can be attributed to the presence of low-molecular-weight phenolic compounds in the rosemary extract, which preferentially interact with the amorphous phase of PLA, restricting segmental mobility and leading to a persistent increase in Tg without significantly altering crystallization behavior.

For PLA/spirulina composites thermally treated for 16 and 32 h, Tg, onset increases from the second to the third DSC heating cycle, and the magnitude of this increase scales with spirulina content. This behavior suggests progressive development of a constrained amorphous interphase upon repeated thermal cycling, likely promoted by the polar biomolecular components of spirulina (e.g., proteins, pigments such as phycocyanin) that can enhance PLA–filler interactions.

Overall, these results indicate that the presence of fillers alters the relative contributions of mobile and constrained amorphous fractions in PLA, resulting in filler-content-dependent changes in Tg and ΔCp. The observed variations are primarily attributed to physical effects related to confinement, interfacial interactions, and thermal history rather than definitive evidence of chemical degradation, which cannot be excluded on the basis of DSC analysis alone.

#### 3.4.2. Cold Crystallization

For the neat PLA samples (Figure S2a), the cold crystallization behavior shows systematic changes with both DSC cycling and prior thermal treatment. Between the second and third DSC heating steps, an increase in both the cold crystallization onset temperature (Tc, onset) and the cold crystallization enthalpy (ΔHc) is observed, indicating enhanced crystallization ability after repeated thermal cycling. In contrast, increasing the duration of the mild thermal treatment at 80 °C (8, 16, and 32 h) leads to a progressive decrease in both Tc, onset (~89 °C) and ΔHc, suggesting that prolonged annealing reduces the amount of crystallizable material, likely due to the development of constrained amorphous regions and/or limited chain degradation that restrict crystal growth.

For PLA samples filled with kelp or spirulina biomass (Figure S2b–e,j–m), both the untreated and non-pre-annealed samples show an increase in the cold-crystallization onset temperature (Tc, onset) and the cold-crystallization enthalpy (ΔHc) between the second and third DSC heating cycles, indicating enhanced crystallization upon repeated thermal cycling. For thermally treated samples (8, 16, and 32 h at 80 °C), Tc, onset, and ΔHc increase with increasing kelp or spirulina content, suggesting a filler-dependent effect on the crystallization behavior after annealing.

In contrast, PLA samples containing rosemary extract (Figure S2f–i) exhibit a different trend. Tc, onset increases between the second and third DSC heating cycles for all compositions. For untreated and 8 h thermally treated samples, both Tc, onset, and ΔHc decrease when rosemary extract is added up to 0.5 wt% compared with neat PLA, while higher loadings (above 0.5 wt%, up to 3 wt%) lead to increasing Tc, onset, and ΔHc values. This behavior changes for samples thermally treated for 16 h or longer, indicating that prolonged annealing alters the influence of rosemary extract on cold-crystallization behavior.

#### 3.4.3. Melting

The melting behavior of the neat PLA samples shows a systematic dependence on prior thermal treatment and DSC cycling (Figure S3a). During the first DSC heating cycle, the melting onset temperature is observed at approximately 170 °C and increases slightly with increasing duration of the mild thermal treatment at 80 °C (8, 16, and 32 h), indicating the formation of more stable or better-organized crystalline domains. At the same time, the melting enthalpy (ΔHm, endothermic) decreases with increasing thermal treatment time, suggesting a reduction in the overall crystalline fraction or the development of less perfect crystals. In the second and third DSC heating cycles, the melting onset temperature is lower, around ~166 °C, reflecting the removal of the prior thermal history after the first heating–cooling cycle. A small increase of approximately 1–2 °C in the melting onset temperature is observed in the third heating cycle, indicating minor reorganization or perfection of crystalline structures upon repeated thermal cycling.

For the filled PLA samples, the overall trend of the melting onset temperature follows that observed for neat PLA (Figure S3b–m). In thermally treated PLA containing kelp biomass, the melting onset temperature decreases with increasing treatment duration up to 16 h, suggesting the formation of less stable or more heterogeneous crystalline domains. However, for samples treated for 32 h, the melting onset temperature increases again, indicating partial reorganization or stabilization of crystalline structures upon prolonged thermal exposure.

In thermally treated PLA containing kelp biomass, the melting onset temperature decreases with increasing filler content up to 1 wt%, indicating reduced crystal stability at low filler loadings. However, at higher kelp contents (above 1 wt% and up to 3 wt%), the melting onset temperature increases again, suggesting partial stabilization or reorganization of crystalline domains at higher filler concentrations. PLA samples filled with rosemary extract exhibit an opposite trend in melting onset temperature compared with kelp-filled systems, with the onset temperature increasing at low extract contents and decreasing at higher loadings. In contrast, for thermally treated PLA containing spirulina biomass, the melting onset temperature decreases monotonically with increasing filler content, showing an inverse relationship with spirulina concentration. These distinct behaviors highlight the different roles of the fillers, reflecting differences in their chemical composition and interfacial interactions with the PLA matrix, which influence crystal stability in different ways.

#### 3.4.4. Melt Crystallization

The DSC analysis of unfilled PLA samples (Figure S4a) shows that the melt-crystallization onset temperature remains essentially unchanged with increasing duration of the thermal treatment but shifts to slightly lower values during the second cooling step compared with the first. In contrast, the melt-crystallization enthalpy (ΔHmc) increases progressively with longer thermal treatment during sample preparation, indicating enhanced crystallization upon cooling as a result of prior thermal conditioning. The decrease in onset temperature between successive cooling steps suggests that, after the first heating–cooling cycle, crystal nucleation occurs more readily, allowing crystallization to start at lower temperatures while producing a larger crystalline fraction.

For PLA samples filled with kelp biomass, the melt-crystallization behavior follows a trend similar to that of neat PLA. In several cases, however, the crystallization peak observed during the second cooling step becomes significantly broadened, leading to reduced ΔHmc values; in some samples, the crystallization peak is no longer detectable in the second cooling cycle. This behavior suggests a reduced crystallization efficiency and increased heterogeneity in crystal formation after repeated thermal cycling.

In contrast, PLA samples containing rosemary extract show a systematic decrease in both the melt-crystallization onset temperature and ΔHmc with increasing extract content. This trend was consistently observed during both the first and second cooling steps and became more pronounced for samples thermally treated for 16 and 32 h. A similar pattern is also observed for PLA samples filled with spirulina biomass, particularly after longer thermal treatments (16 and 32 h). These results indicate that rosemary extract and spirulina biomass increasingly hinder melt crystallization at higher loadings, likely due to enhanced interfacial constraints and reduced chain mobility during cooling.

## 4. Discussion

Recent research highlights a clear shift toward fully bio-based PLA formulations in which both plasticizers and stabilizing additives are derived from natural sources. Studies on filled PLA demonstrated that epoxidized and maleinized vegetable oils—such as Brazil nut, hemp seed, and corn oils—effectively plasticize PLA, improving ductility and processability while maintaining its biodegradable character, although plasticization may also increase chain mobility and susceptibility to thermo-oxidative degradation [44,45]. Furthermore, the importance of stabilizing strategies to counteract such degradation phenomena was emphasized [44,46]. In this context, natural antioxidants have emerged as promising multifunctional additives in the effort to enhance the thermal stability and lifespan of biodegradable polymers without compromising sustainability [47]. Specifically for PLA, Valero, L. et al. (2024) demonstrated that vegetal polyphenol extracts can significantly retard oxidative degradation, enabling fully bio-based stabilized formulations [48]. Moreover, Stoyanova, N. et al. (2023) showed that incorporating Melissa officinalis extract into electrospun PLA not only provided strong antioxidant activity but also added functional bioactivity, expanding potential biomedical applications [49]. Overall, synthesizing these studies indicates that combining vegetable oil-based plasticizers with natural antioxidant systems represents a coherent strategy to improve PLA flexibility while preserving or even enhancing its oxidative stability, thereby advancing the development of high-performance, fully sustainable bioplastic materials.

**Figure 10:**
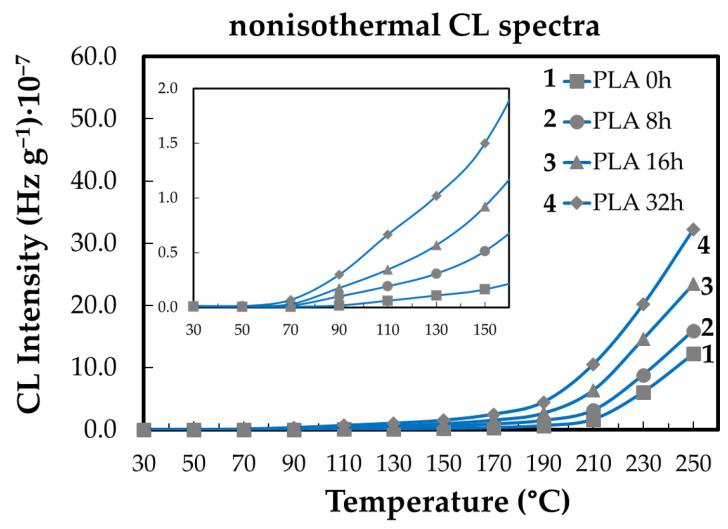
The nonisothermal CL spectra ascribed to neat PLA degraded at various times (heating rate: 20 °C min^−1^). Heating period: (1) non; (2) one cycle of 8 h; (3) two cycles of 8 h; (4) four cycles of 8 h.

The lifetime of polymeric materials is influenced by both their intrinsic structural characteristics and external conditions that provide the energy required to initiate oxidative degradation processes [35,50]. Antioxidants contribute to improved durability by interrupting oxidation chain reactions, thereby extending the service life and widening the application range of polymer products [36,51]. In PLA, degradation predominantly occurs through random chain scission, resulting in molecular fragmentation via oxidative and depolymerization pathways [5,38,52,53]. Chemiluminescence (CL) analysis offers a sensitive approach for monitoring these thermo-oxidative processes through photon emission during thermal exposure. Non-isothermal CL measurements reveal that oxidative degradation of PLA becomes significant at temperatures above approximately 50 °C, as illustrated in Figure 6 and Figure 10, reflecting the temperature-driven activation of degradation kinetics.

**Figure 11:**
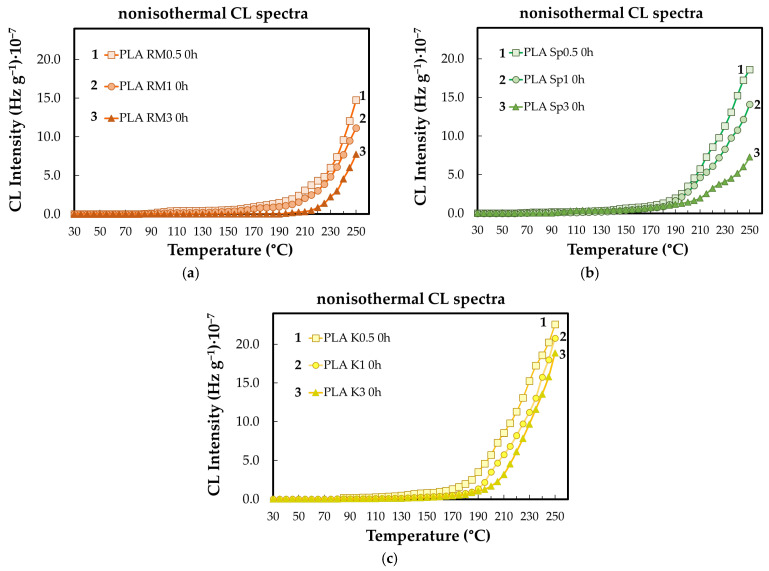
The nonisothermal CL spectra recorded on the modified PLA samples (Heating rate: 5 °C min^−1^). Additive: (**a**) RM; (**b**) SP; (**c**) K. Additive loading: (1) 0.5%; (2) 1 wt%; (3) 3 wt%.

- The lifetime of polymeric materials is governed by both intrinsic structural factors and external conditions that supply the energy required for oxidative degradation [35,50].
- Antioxidants enhance durability by interrupting oxidation chain reactions, thereby extending material service life and broadening application potential [36,51].
- In PLA, degradation proceeds mainly through random chain scission, leading to molecular fragmentation via oxidative and depolymerization pathways [5,38,52,53].
- Chemiluminescence (CL) analysis provides direct insight into thermo-oxidative degradation by monitoring photon emission during thermal exposure.
- Non-isothermal CL measurements show that significant oxidative degradation of PLA is initiated at temperatures above approximately 50 °C, as evidenced in Figure 6 and Figure 10, consistent with temperature-activated degradation kinetics.
- CL-derived oxidation induction times (OITs) reveal distinct stabilization efficiencies among the additives, with rosemary extract providing the longest OITs and kelp-based fillers the shortest, reflecting different abilities to delay oxidation onset.
- These trends are consistent with DSC and FTIR results: rosemary extract increases Tg and limits hydroxyl-group formation, while biomass fillers mainly influence crystallization behavior and chain constraints through heterogeneous interfacial regions.
- Increasing the additive content up to 3 wt% enhances stability (Figure 11), confirming the effectiveness of these mechanisms within the investigated concentration range.
- Overall, the combined CL, DSC, and FTIR analyses indicate that the natural additives improve PLA thermal stability primarily by slowing oxidation kinetics and modifying amorphous-phase mobility, rather than by completely suppressing depolymerization.
- The stabilizing efficiency is therefore governed by a combination of antioxidant activity and polymer–filler interfacial interactions under moderate to severe thermal conditions [54,55,56].

Although all investigated fillers influence the thermal stability of PLA, their stabilization mechanisms differ fundamentally. Rosemary extract acts primarily through a chemical antioxidant mechanism, as it contains phenolic compounds capable of efficiently scavenging peroxy and alkyl radicals, thereby interrupting oxidation chain reactions and delaying oxidation onset. This behavior is reflected in the extended oxidation induction times, increased Tg, and reduced formation of oxidation-related hydroxyl groups. In contrast, algal biomasses such as spirulina and kelp contribute mainly through physical and interfacial effects rather than direct radical scavenging. Their incorporation introduces heterogeneous regions within the PLA matrix that modify chain mobility, crystallization behavior, and oxygen diffusion pathways, resulting in a more moderate and formulation-dependent stabilization effect. Consequently, while rosemary extract provides a direct and efficient antioxidative response, algal fillers primarily mitigate degradation by altering the morphology and kinetics of oxidation rather than by suppressing radical formation itself.

## 5. Conclusions

The results demonstrate that PLA degradation under the investigated conditions is governed primarily by thermo-oxidative radical processes, with hydrolytic cleavage of ester groups contributing at advanced stages of degradation when oxidation products and moisture are present. Non-isothermal CL measurements indicate that oxidative activity becomes significant above approximately **50 °C**, while prolonged thermal exposure at **80 °C (8–32 h)** induces measurable pre-ageing effects without triggering extensive depolymerization.

The incorporation of natural additives at loadings up to **3 wt%** significantly influences PLA stability. Among the studied fillers, **rosemary extract exhibits the highest stabilization efficiency**, as evidenced by the **longest oxidation induction times (OITs)**, increased **glass-transition temperature (Tg)**, and suppressed formation of oxidation-related hydroxyl groups. In contrast, spirulina- and kelp-based fillers mainly affect chain mobility and morphology through interfacial interactions, resulting in more moderate and formulation-dependent stabilization effects.

From an application perspective, the combined CL, DSC, and FTIR results indicate that natural additives can effectively **delay oxidation kinetics and improve thermal resistance** of PLA under moderate and severe thermal conditions relevant to processing and use. In particular, rosemary-modified PLA shows strong potential for **food-packaging applications**, where enhanced oxidative stability, controlled ageing behavior, and the use of naturally derived stabilizers are critical for extending service life and maintaining material safety.

## Figures and Tables

**Table 1:** IR main absorption bands of PLA tested samples [40,41,42,43].

Wavenumber (cm^−1^)	Assignment
3500–3200	weak/broad O–H stretching—terminal –OH, moisture), possible oxidation intermediates
2995–2945	–CH_3_ asymmetric stretching
2945–2880	–CH_3_ symmetric stretching
1749	very strong C=O stretching of ester group (signature peak of PLA)
1454	CH_3_ asymmetric bending—reference peak
1385–1360	CH_3_ symmetric bending
1265	C–O–C stretching (ester)
1210, 1180	C–O stretching
1130, 1080	C–O–C asymmetric stretching
1042	C–O stretching of the backbone
957	C–O–C stretching and skeletal vibrations of the polymer backbone associated with the amorphous phase of PLA
920	C–COO stretching vibrations associated with the crystalline phase of PLA
870	C–COO stretching/CH_3_ rocking
754	PLA skeletal vibrations

## Data Availability

The raw data supporting the conclusions of this article will be made available by the authors on request.

## Supplementary Materials

The following supporting information can be downloaded at: https://www.mdpi.com/article/10.3390/polym18040504/s1, Figure S1. Glass transition modifications recorded for samples with: (a) no filler, filled with kelp biomass filler, (b) no thermal treatment, (c) 8 h thermal treatment, (d) 16 h thermal treatment, (e) 32 h thermal treatment, filled with rosemary extract, (f) no thermal treatment, (g) 8 h thermal treatment, (h) 16 h thermal treatment, (i) 32 h thermal treatment, and filled with spirulina biomass, (j) no thermal treatment, (k) 8 h thermal treatment, (l) 16 h thermal treatment, (m) 32 h thermal treatment; Figure S2. Cold crystallization modifications recorded for samples with: (a) no filler, filled with kelp biomass filler, (b) no thermal treatment, (c) 8 h thermal treatment, (d) 16 h thermal treatment, (e) 32 h thermal treatment, filled with rosemary extract, (f) no thermal treatment, (g) 8 h thermal treatment, (h) 16 h thermal treatment, (i) 32 h thermal treatment, and filled with spirulina biomass, (j) no thermal treatment, (k) 8 h thermal treatment, (l) 16 h thermal treatment, (m) 32 h thermal treatment; Figure S3. Melting modifications in DSC analysis recorded for samples with: (a) no filler, filled with kelp biomass filler, (b) no thermal treatment, (c) 8 h thermal treatment, (d) 16 h thermal treatment, (e) 32 h thermal treatment, filled with rosemary extract, (f) no thermal treatment, (g) 8 h thermal treatment, (h) 16 h thermal treatment, (i) 32 h thermal treatment, and filled with spirulina biomass, (j) no thermal treatment, (k) 8 h thermal treatment, (l) 16 h thermal treatment, (m) 32 h thermal treatment; Figure S4. Melt crystallization recorded for samples with: (a) no filler, filled with kelp biomass filler, (b) no thermal treatment, (c) 8 h thermal treatment, (d) 16 h thermal treatment, (e) 32 h thermal treatment, filled with rosemary extract, (f) no thermal treatment, (g) 8 h thermal treatment, (h) 16 h thermal treatment, (i) 32 h thermal treatment, and filled with spirulina biomass, (j) no thermal treatment, (k) 8 h thermal treatment, (l) 16 h thermal treatment, (m) 32 h thermal treatment; Table S1. Correlation between the sample preparation and the notations provided in the manuscript.

## Abbreviations

The following abbreviations are used in this manuscript:

AO	antioxidant
CL	chemiluminescence
Cp	heat capacity
DSC	Differential scanning calorimetry
FTIR	Fourier Transform Infrared Spectroscopy
Hc	cold crystallization enthalpy
Hm	melting enthalpy
Hmc	melt crystallization enthalpy
K	kelp (*Ascophyllum nodosum*)
OIT	oxidation induction time
OOT	onset oxidation temperature
PLA	poly(lactic) acid
RM	rosemary (*Rosmarinus officinalis*)
SIS	styrene–isoprene–styrene
SP	spirulina (*Arthrosipra platensis*)
Tg	glass transition temperature
Tc	crystallization temperature
v_ox_	oxidation rate
