# Supplementary material for: Oxidation Strength of PLA Filled with Algal Biomass and Rosemary Extract Powders for Food-Safe Handling
Source: Polymers (Basel). 2026 Feb 18;18(4):504. doi: 10.3390/polym18040504 (PMC12943856; doi:10.3390/polym18040504)
Supplement: Supplementary file 1 [file polymers-18-00504-s001.zip › polymers-4130916-supplementary.pdf]

polymers

MDPI

Supplementary material

Oxidation Strength of PLA Filled with Algal Biomass and Rosemary Extract Powders for Food-Safe Handling

Traian Zaharescu[1], Marius Bumbac[2,*], Cristina Mihaela Nicolescu[3,*], Aurora Craciun[4], Radu Mirea[5]

[1] Radiochemistry Center, National Institute for R&D in Electrical Engineering ICPE-CA Bucharest, 030138 Bucharest, Romania; traian.zaharescu@icpe-ca.ro
[2] Faculty of Science and Arts, Valahia University of Targoviste, 130004 Targoviste, Romania
[3] Institute of Multidisciplinary Research for Science and Technology, Valahia University of Targoviste, 130004 Targoviste, Romania
[4] Faculty of Medicine, University Ovidius Constanta, 900527 Constanta, Romania; aurora.craciun@365.univ-ovidius.ro
[5] Romanian Research and Development Institute for Gas Turbines—COMOTI, 061125 Bucharest, Romania; radu.mirea@comoti.ro
* Correspondence: marius.bumbac@valahia.ro (M.B.); cristina.nicolescu@valahia.ro (C.M.N.); Tel.: +40-721219270 (M.B.); +40-722246416 (C.M.N.)

Table S1. Correlation between the sample preparation and the notations provided in the manuscript

Notation	Thermal treatment time (hours)	Filler added	Percentage of the filler added
PLA 0h	0	no	0
PLA 8h	8	no	0
PLA 16h	16	no	0
PLA 32h	32	no	0
PLA K0.5 0h	0	Ascophyllum nodosum (kelp) biomass	0.5
PLA K1 0h	0	Ascophyllum nodosum (kelp) biomass	1
PLA K3 0h	0	Ascophyllum nodosum (kelp) biomass	3
PLA 0.5K 8h	8	Ascophyllum nodosum (kelp) biomass	0.5
PLA K1 8h	8	Ascophyllum nodosum (kelp) biomass	1
PLA K3 8h	8	Ascophyllum nodosum (kelp) biomass	3
PLA K0.5 16h	16	Ascophyllum nodosum (kelp) biomass	0.5
PLA K1 16h	16	Ascophyllum nodosum (kelp) biomass	1
PLA K3 16h	16	Ascophyllum nodosum (kelp) biomass	3
PLA K0.5 32h	32	Ascophyllum nodosum (kelp) biomass	0.5

PLA K1 32h	32	Ascophyllum nodosum (kelp) biomass	1
PLA K3 32h	32	Ascophyllum nodosum (kelp) biomass	3
PLA RM0.5 0h	0	Rosmarinus officinalis (rosemary) extract	0.5
PLA RM1 0h	0	Rosmarinus officinalis (rosemary) extract	1
PLA RM3 0h	0	Rosmarinus officinalis (rosemary) extract	3
PLA RM0.5 8h	8	Rosmarinus officinalis (rosemary) extract	0.5
PLA RM1 8h	8	Rosmarinus officinalis (rosemary) extract	1
PLA RM3 8h	8	Rosmarinus officinalis (rosemary) extract	3
PLA RM0.5 16h	16	Rosmarinus officinalis (rosemary) extract	0.5
PLA RM1 16h	16	Rosmarinus officinalis (rosemary) extract	1
PLA RM3 16h	16	Rosmarinus officinalis (rosemary) extract	3
PLA RM0.5 32h	32	Rosmarinus officinalis (rosemary) extract	0.5
PLA RM1 32h	32	Rosmarinus officinalis (rosemary) extract	1
PLA RM3 32h	32	Rosmarinus officinalis (rosemary) extract	3
PLA Sp0.5 0h	0	Arthrospira platensis (spirulina) biomass	0.5
PLA Sp1 0h	0	Arthrospira platensis (spirulina) biomass	1
PLA Sp3 0h	0	Arthrospira platensis (spirulina) biomass	3
PLA Sp0.5 8h	8	Arthrospira platensis (spirulina) biomass	0.5
PLA Sp1 8h	8	Arthrospira platensis (spirulina) biomass	1
PLA Sp3 8h	8	Arthrospira platensis (spirulina) biomass	3
PLA Sp0.5 16h	16	Arthrospira platensis (spirulina) biomass	0.5
PLA Sp1 16h	16	Arthrospira platensis (spirulina) biomass	1
PLA Sp3 16h	16	Arthrospira platensis (spirulina) biomass	3
PLA Sp0.5 32h	32	Arthrospira platensis (spirulina) biomass	0.5
PLA Sp1 32h	32	Arthrospira platensis (spirulina) biomass	1
PLA Sp3 32h	32	Arthrospira platensis (spirulina) biomass	3

https://doi.org/10.3390/polym18040504

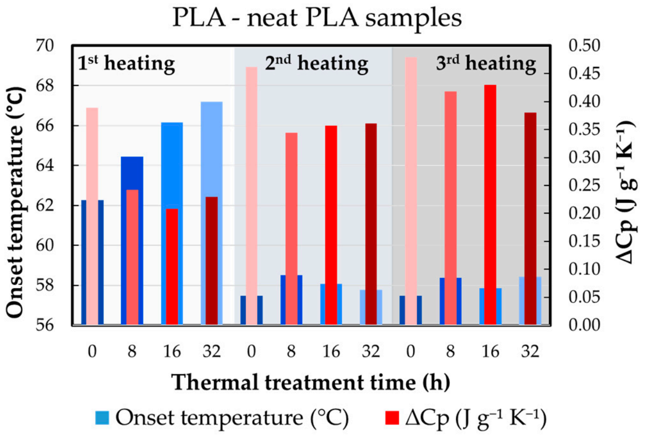

(a)

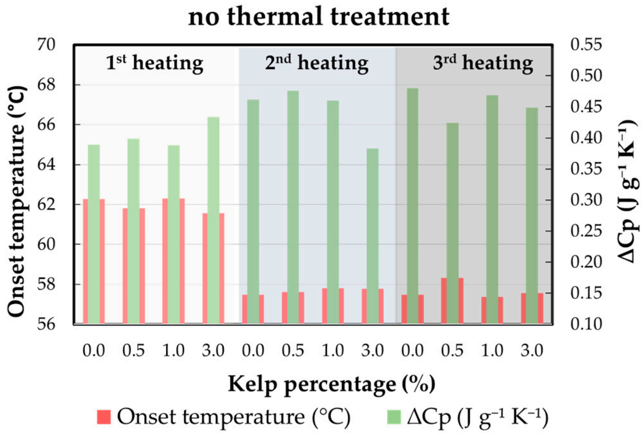

(b)

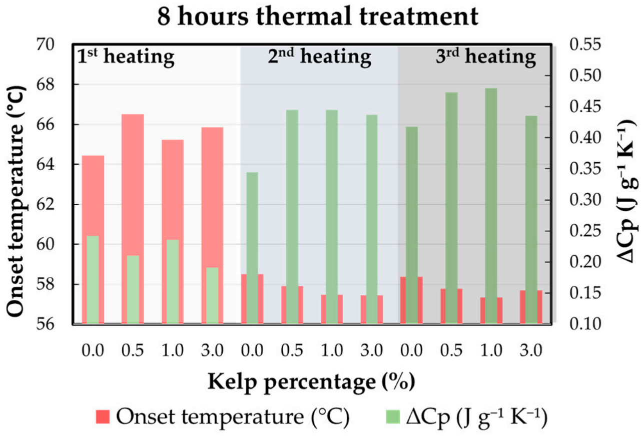

(c)

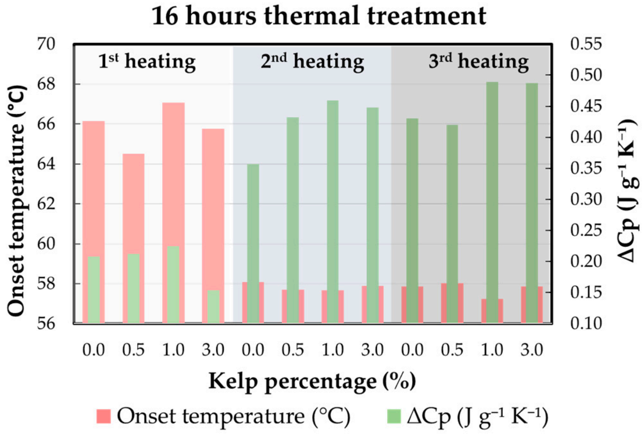

(d)

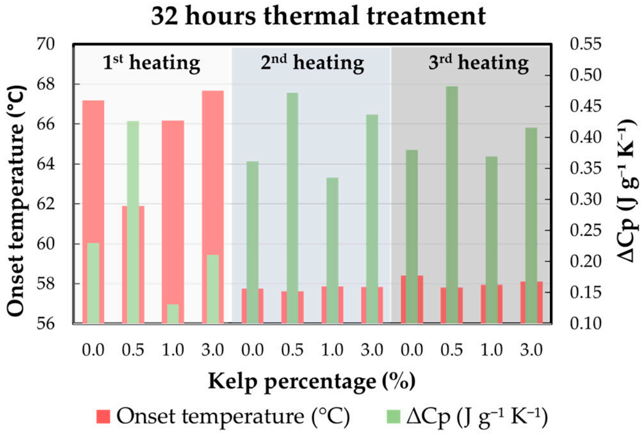

(e)

https://doi.org/10.3390/polym18040504

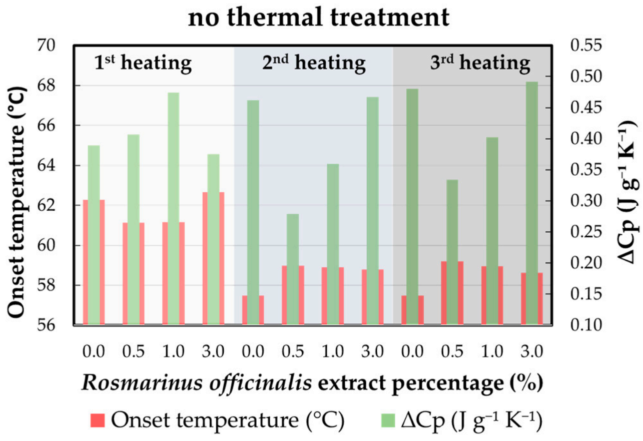

(f)

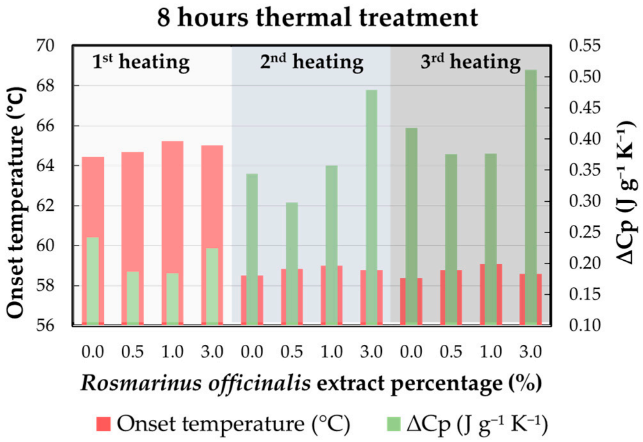

(g)

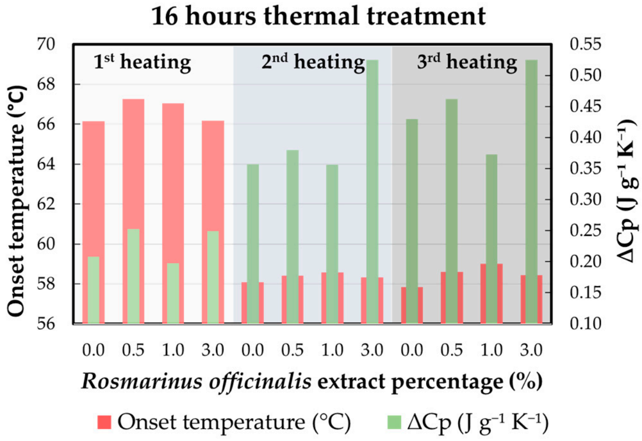

(h)

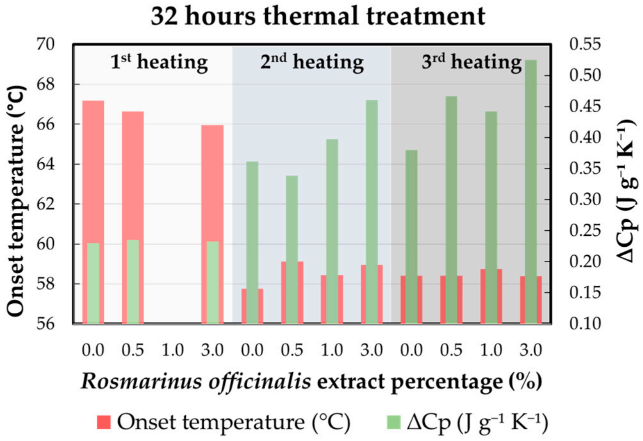

(i)

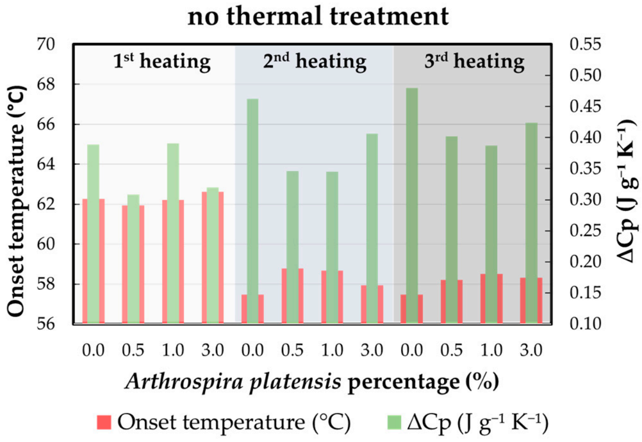

(j)

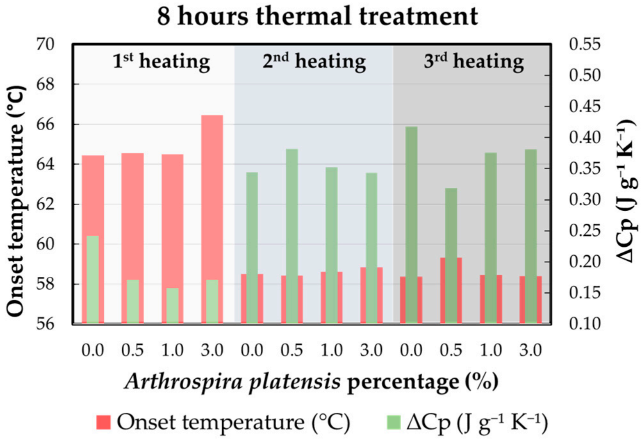

(k)

https://doi.org/10.3390/polym18040504

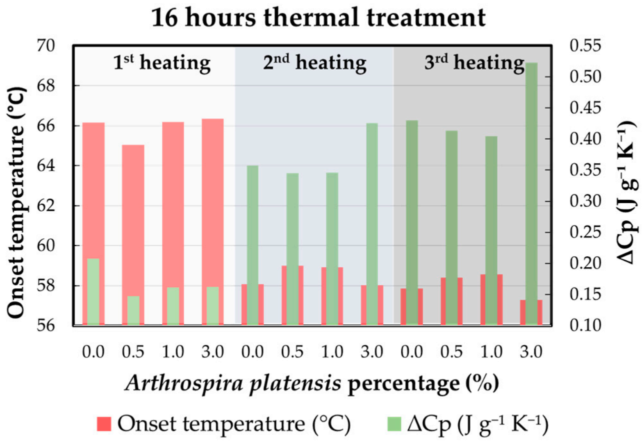

(l)

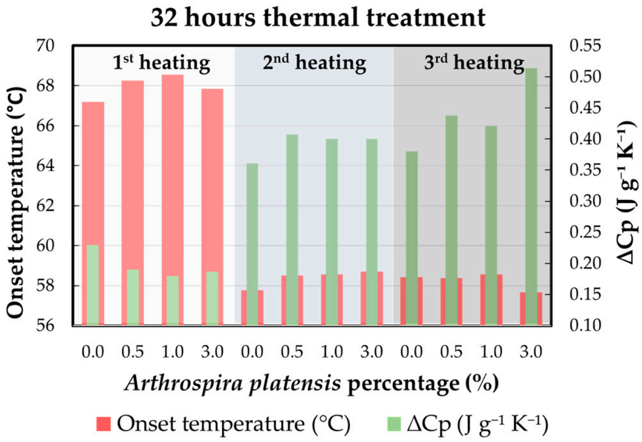

(m)

Figure S1. Glass transition modifications recorded for samples with: a) no filler, filled with kelp biomass filler, b) no thermal treatment, c) 8 hours thermal treatment, d) 16 hours thermal treatment, e) 32 hours thermal treatment, filled with rosemary extract, f) no thermal treatment, g) 8 hours thermal treatment, h) 16 hours thermal treatment, i) 32 hours thermal treatment, and filled with spirulina biomass, j) no thermal treatment, k) 8 hours thermal treatment, l) 16 hours thermal treatment, m) 32 hours thermal treatment

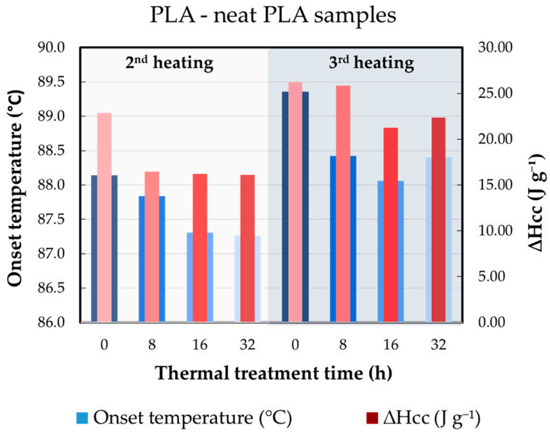

(a)

https://doi.org/10.3390/polym18040504

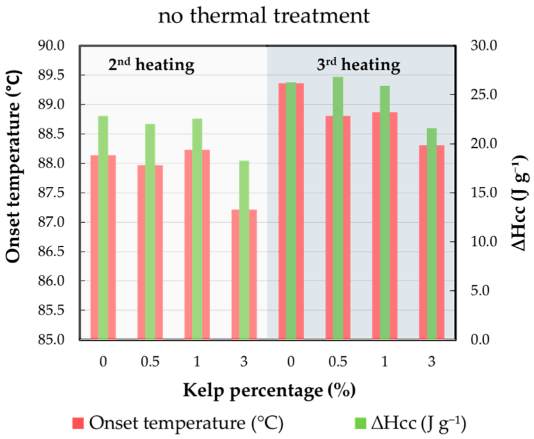
no thermal treatment
2nd heating
3rd heating
Onset temperature (°C)
ΔHcc (J g−1)
Kelp percentage (%)
Onset temperature (°C)
ΔHcc (J g−1)

(b)

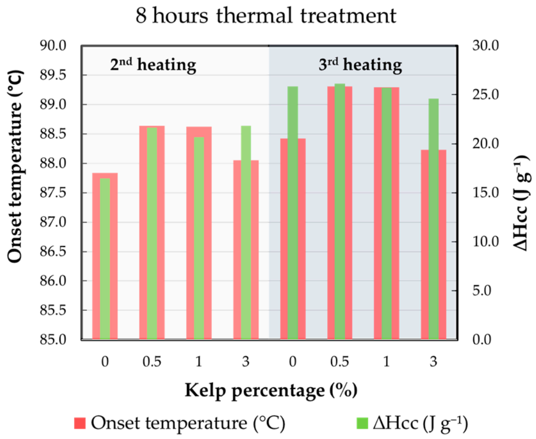
8 hours thermal treatment
2nd heating
3rd heating
Onset temperature (°C)
ΔHcc (J g−1)
Kelp percentage (%)
Onset temperature (°C)
ΔHcc (J g−1)

(c)

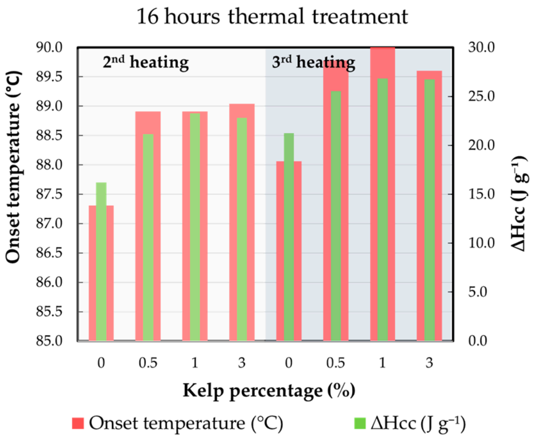
16 hours thermal treatment
2nd heating
3rd heating
Onset temperature (°C)
ΔHcc (J g−1)
Kelp percentage (%)
Onset temperature (°C)
ΔHcc (J g−1)

(d)

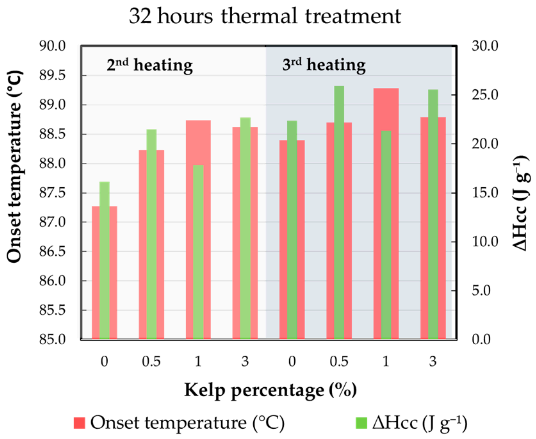
32 hours thermal treatment
2nd heating
3rd heating
Onset temperature (°C)
ΔHcc (J g−1)
Kelp percentage (%)
Onset temperature (°C)
ΔHcc (J g−1)

(e)

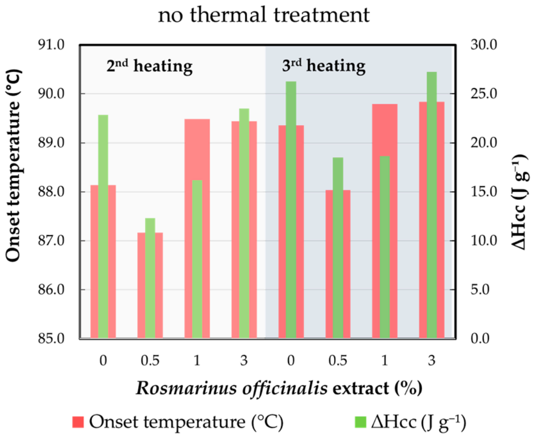
no thermal treatment
2nd heating
3rd heating
Onset temperature (°C)
ΔHcc (J g−1)
Rosmarinus officinalis extract (%)
Onset temperature (°C)
ΔHcc (J g−1)

(f)

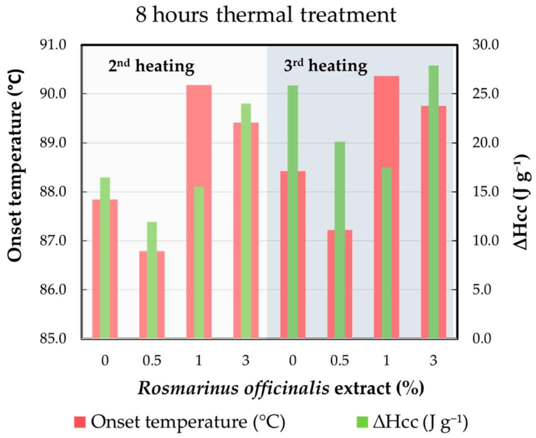
8 hours thermal treatment
2nd heating
3rd heating
Onset temperature (°C)
ΔHcc (J g−1)
Rosmarinus officinalis extract (%)
Onset temperature (°C)
ΔHcc (J g−1)

(g)

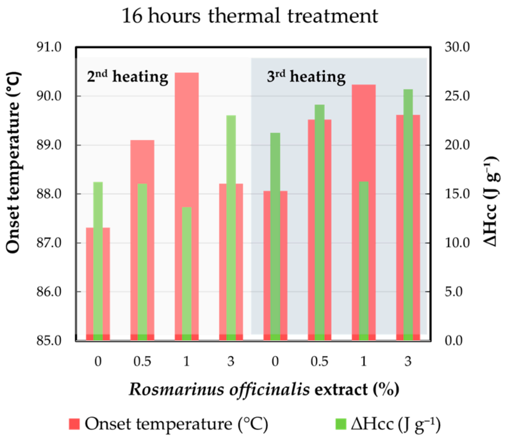

(h)

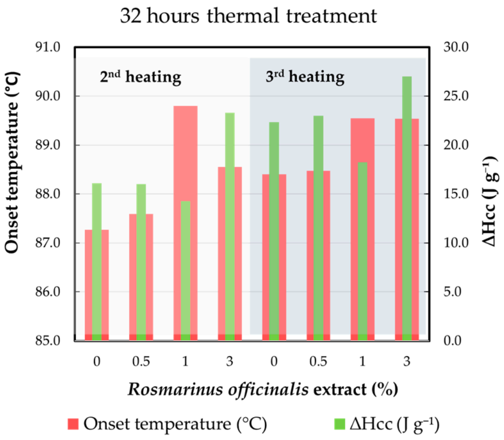

(i)

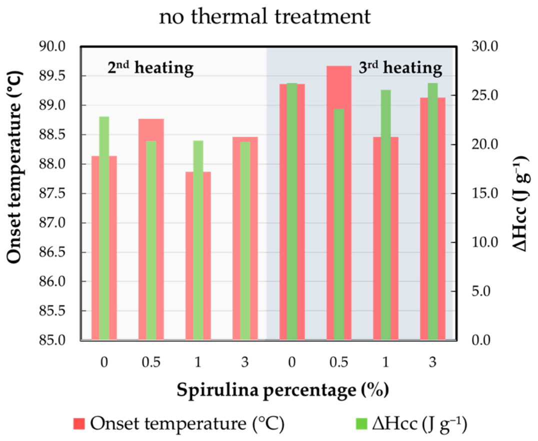

(j)

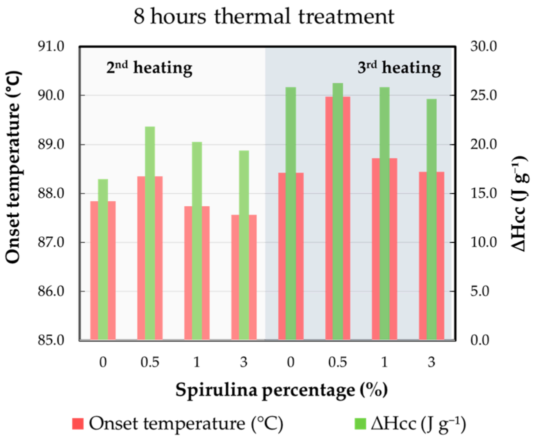

(k)

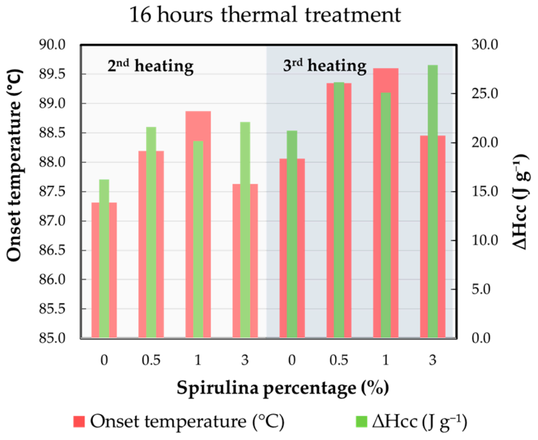

(l)

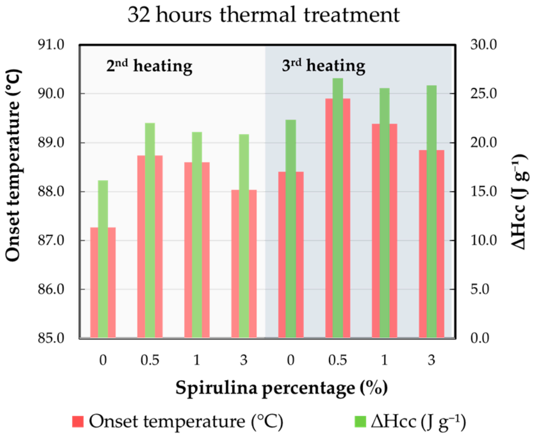

(m)

Figure S2. Cold crystallization modifications recorded for samples with: a) no filler, filled with kelp biomass filler, b) no thermal treatment, c) 8 hours thermal treatment, d) 16 hours thermal treatment,

e) 32 hours thermal treatment, filled with rosemary extract, f) no thermal treatment, g) 8 hours thermal treatment, h) 16 hours thermal treatment, i) 32 hours thermal treatment, and filled with spirulina biomass, j) no thermal treatment, k) 8 hours thermal treatment, l) 16 hours thermal treatment, m) 32 hours thermal treatment

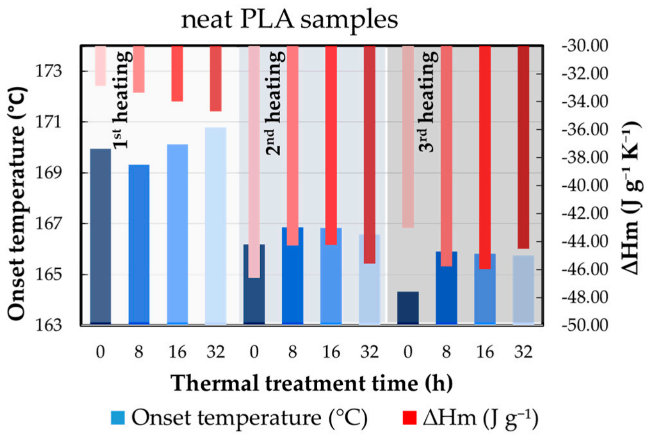

(a)

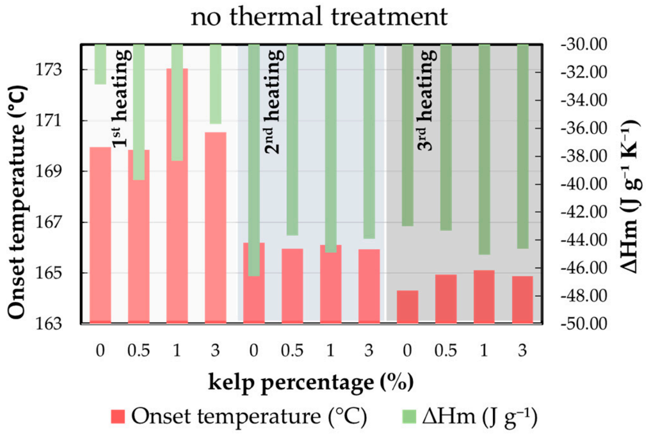

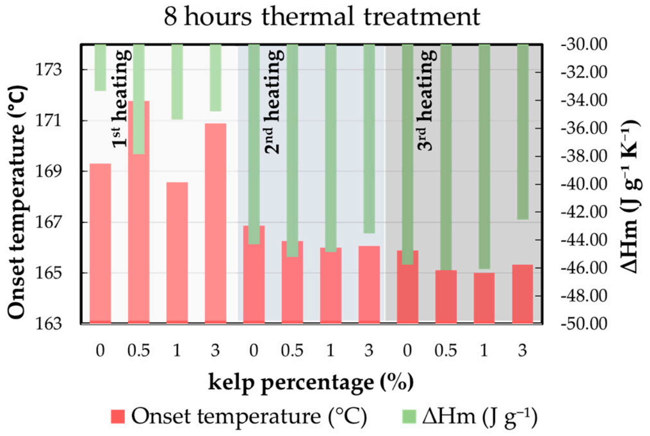

(b) (c)

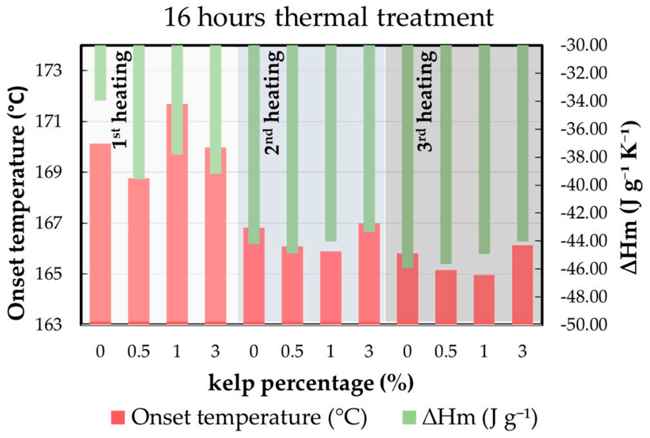

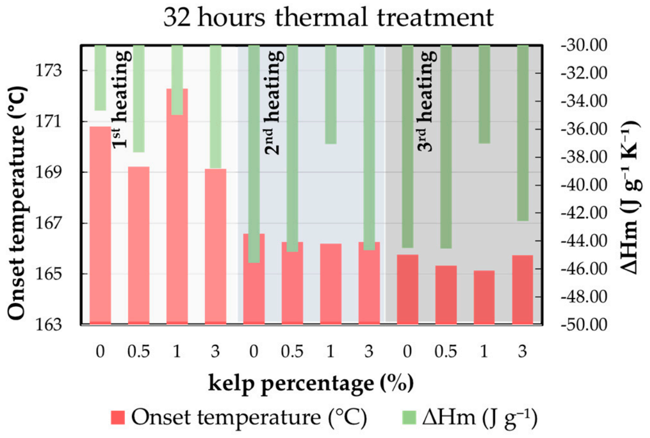

(d) (e)

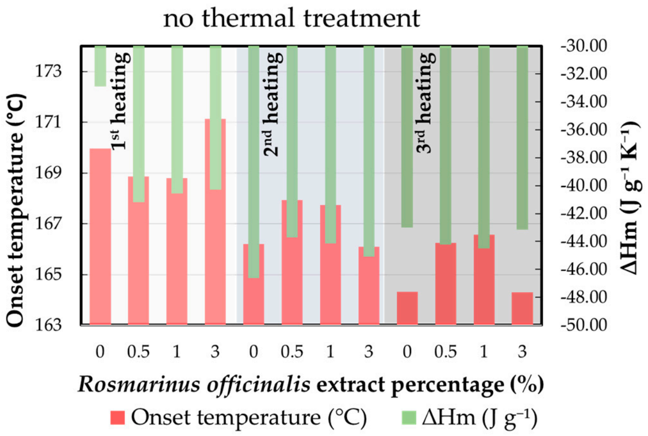
no thermal treatment
Onset temperature (°C)
ΔHm (J g⁻¹ K⁻¹)
1st heating
2nd heating
3rd heating
Rosmarinus officinalis extract percentage (%)
Onset temperature (°C) ΔHm (J g⁻¹)

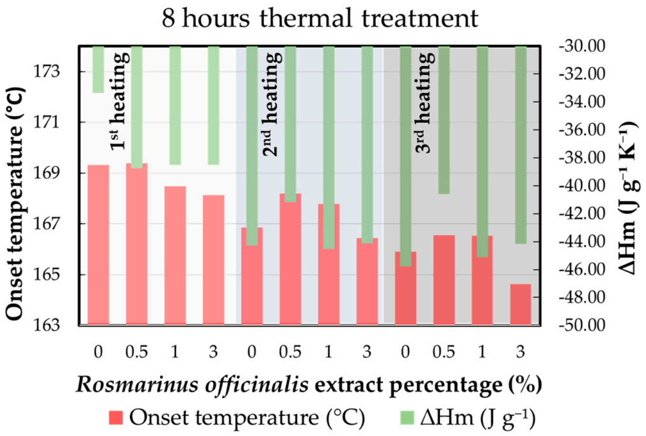
8 hours thermal treatment
Onset temperature (°C)
ΔHm (J g⁻¹ K⁻¹)
1st heating
2nd heating
3rd heating
Rosmarinus officinalis extract percentage (%)
Onset temperature (°C) ΔHm (J g⁻¹)

(f) (g)

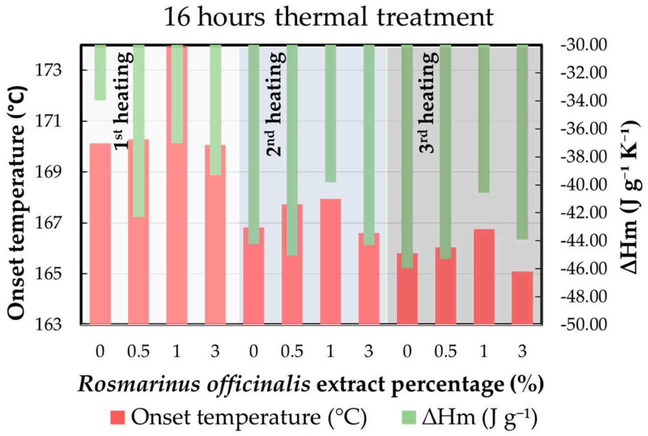
16 hours thermal treatment
Onset temperature (°C)
ΔHm (J g⁻¹ K⁻¹)
1st heating
2nd heating
3rd heating
Rosmarinus officinalis extract percentage (%)
Onset temperature (°C) ΔHm (J g⁻¹)

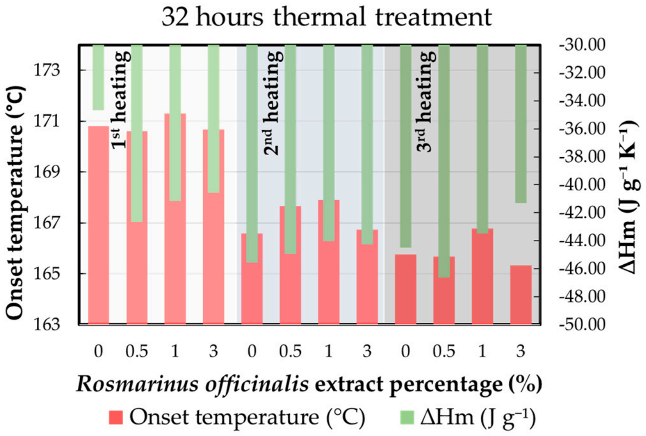
32 hours thermal treatment
Onset temperature (°C)
ΔHm (J g⁻¹ K⁻¹)
1st heating
2nd heating
3rd heating
Rosmarinus officinalis extract percentage (%)
Onset temperature (°C) ΔHm (J g⁻¹)

(h) (i)

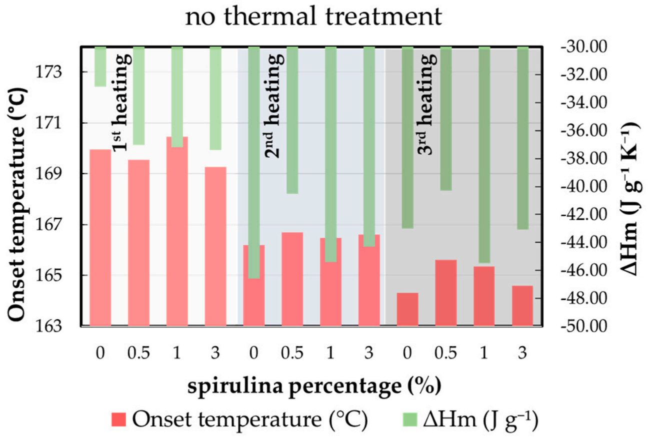
no thermal treatment
Onset temperature (°C)
ΔHm (J g⁻¹ K⁻¹)
1st heating
2nd heating
3rd heating
spirulina percentage (%)
Onset temperature (°C) ΔHm (J g⁻¹)

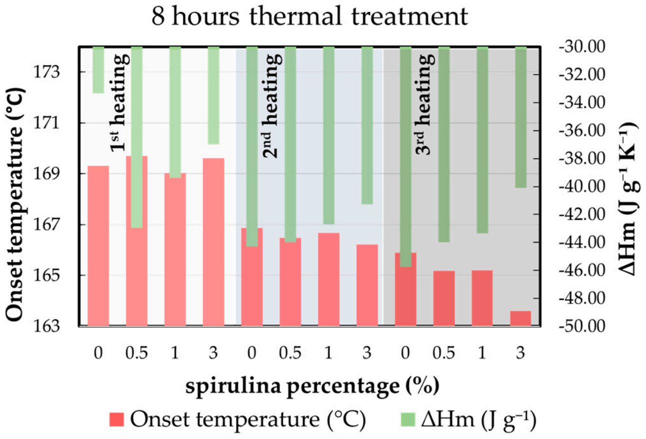
8 hours thermal treatment
Onset temperature (°C)
ΔHm (J g⁻¹ K⁻¹)
1st heating
2nd heating
3rd heating
spirulina percentage (%)
Onset temperature (°C) ΔHm (J g⁻¹)

(j) (k)

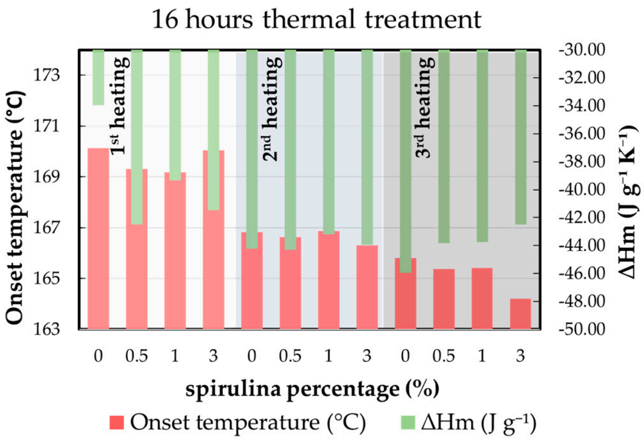

(l)

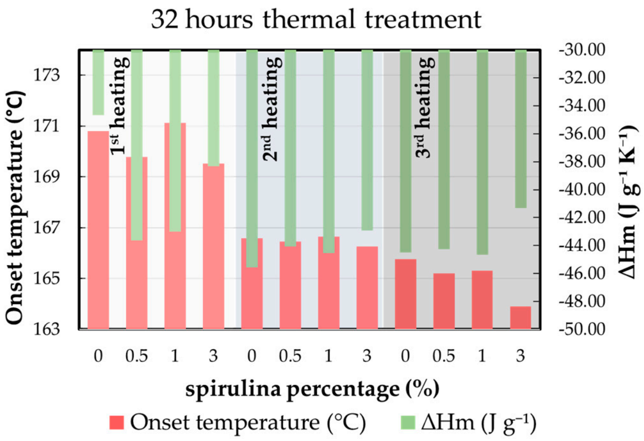

(m)

Figure S3. Melting modifications in DSC analysis recorded for samples with: a) no filler, filled with kelp biomass filler, b) no thermal treatment, c) 8 hours thermal treatment, d) 16 hours thermal treatment, e) 32 hours thermal treatment, filled with rosemary extract, f) no thermal treatment, g) 8 hours thermal treatment, h) 16 hours thermal treatment, i) 32 hours thermal treatment, and filled with spirulina biomass, j) no thermal treatment, k) 8 hours thermal treatment, l) 16 hours thermal treatment, m) 32 hours thermal treatment

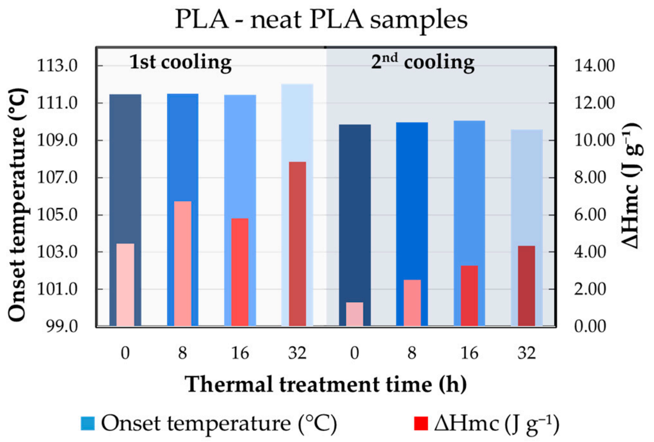

(a)

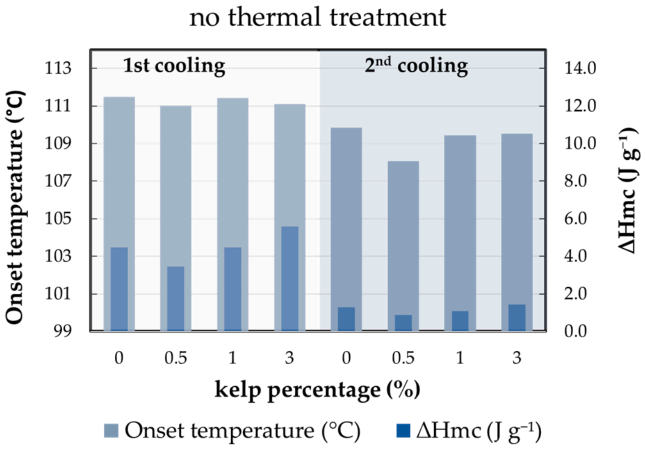
no thermal treatment
1st cooling
2nd cooling
Onset temperature (°C)
ΔHmc (J g−1)
113
111
109
107
105
103
101
99
14.0
12.0
10.0
8.0
6.0
4.0
2.0
0.0
0 0.5 1 3 0 0.5 1 3
kelp percentage (%)
Onset temperature (°C) ΔHmc (J g−1)

(b)

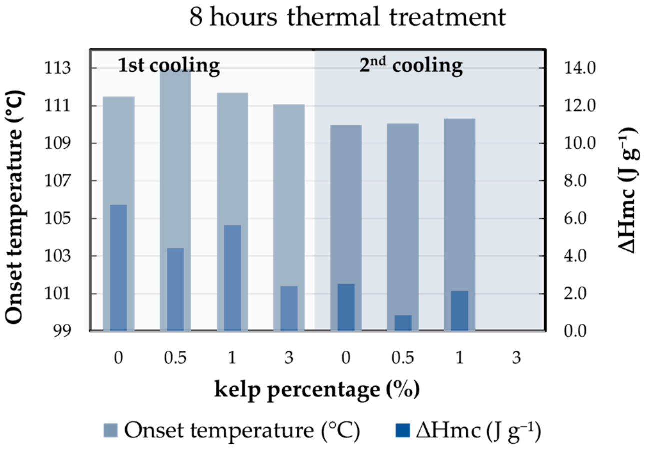
8 hours thermal treatment
1st cooling
2nd cooling
Onset temperature (°C)
ΔHmc (J g−1)
0 0.5 1 3 0 0.5 1 3
kelp percentage (%)
Onset temperature (°C) ΔHmc (J g−1)

(c)

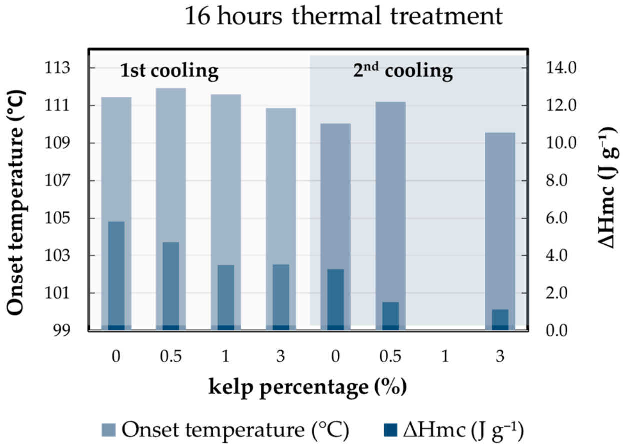
16 hours thermal treatment
1st cooling
2nd cooling
Onset temperature (°C)
ΔHmc (J g−1)
0 0.5 1 3 0 0.5 1 3
kelp percentage (%)
Onset temperature (°C) ΔHmc (J g−1)

(d)

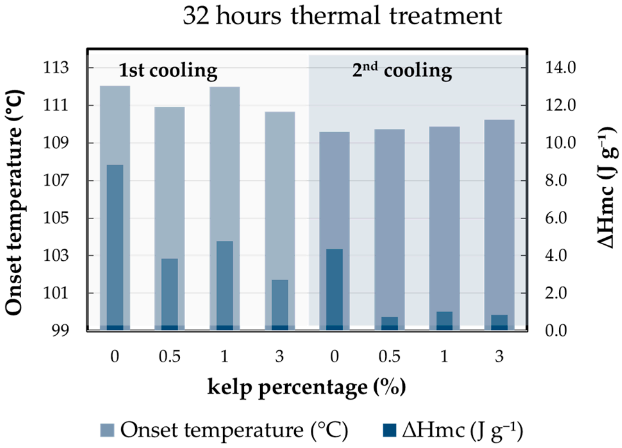
32 hours thermal treatment
1st cooling
2nd cooling
Onset temperature (°C)
ΔHmc (J g−1)
0 0.5 1 3 0 0.5 1 3
kelp percentage (%)
Onset temperature (°C) ΔHmc (J g−1)

(e)

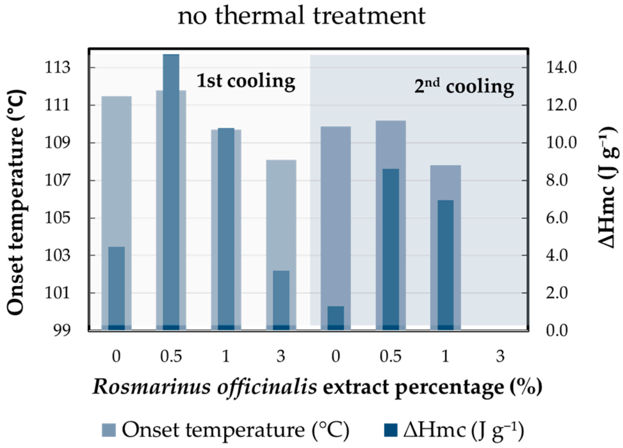
no thermal treatment
1st cooling
2nd cooling
Onset temperature (°C)
ΔHmc (J g−1)
0 0.5 1 3 0 0.5 1 3
Rosmarinus officinalis extract percentage (%)
Onset temperature (°C) ΔHmc (J g−1)

(f)

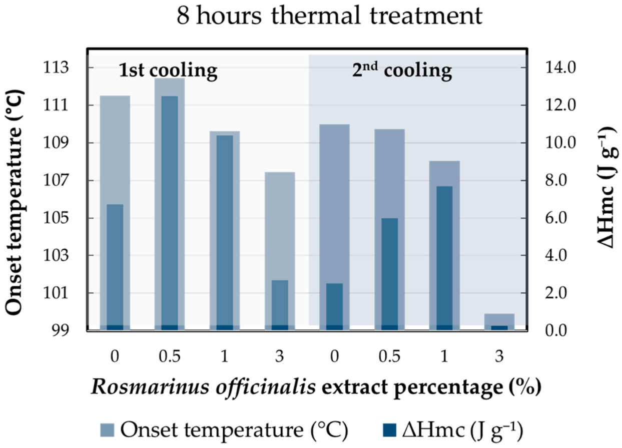
8 hours thermal treatment
1st cooling
2nd cooling
Onset temperature (°C)
ΔHmc (J g−1)
0 0.5 1 3 0 0.5 1 3
Rosmarinus officinalis extract percentage (%)
Onset temperature (°C) ΔHmc (J g−1)

(g)

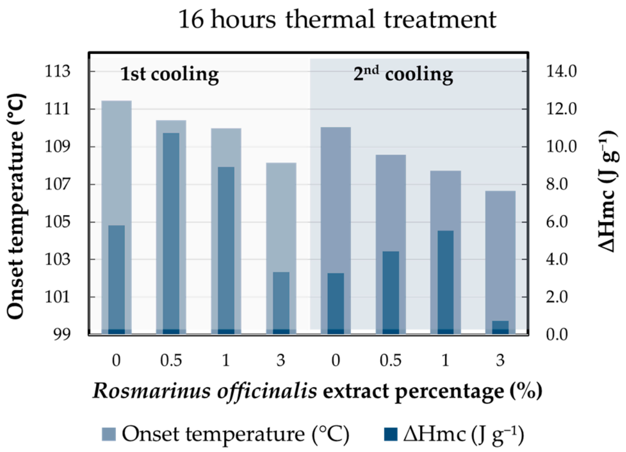

(h)

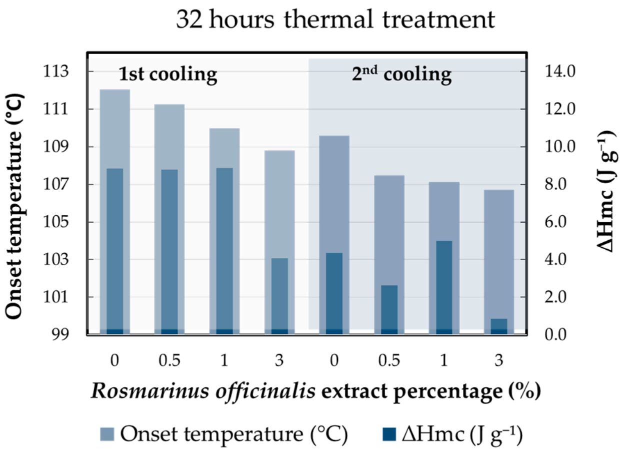

(i)

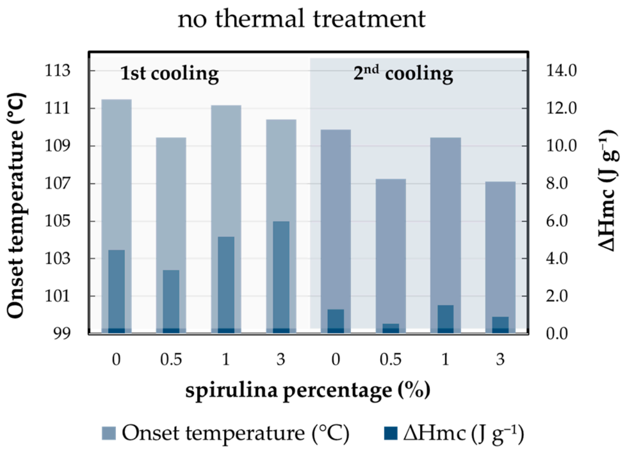

(j)

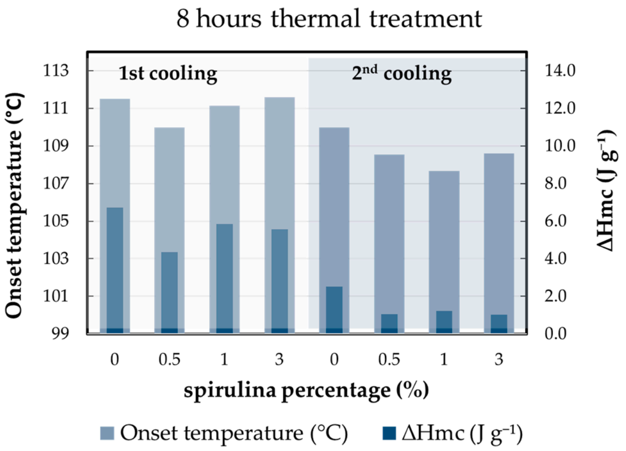

(k)

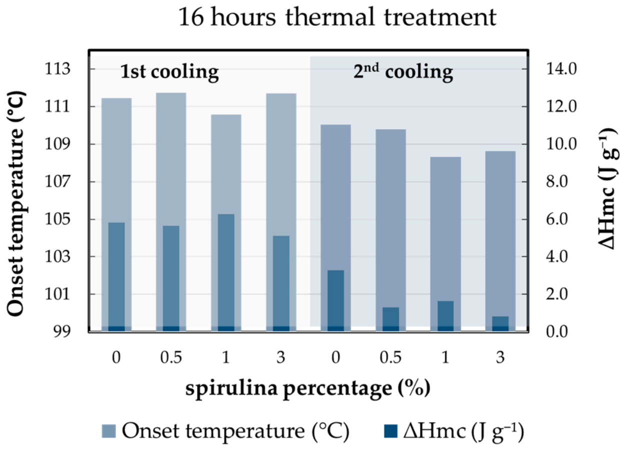

(l)

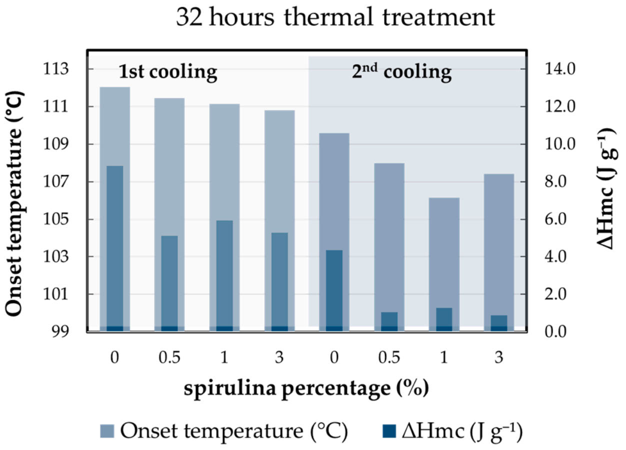

(m)

Figure S4. Melt crystallization recorded for samples with: a) no filler, filled with kelp biomass filler, b) no thermal treatment, c) 8 hours thermal treatment, d) 16 hours thermal treatment, e) 32 hours

https://doi.org/10.3390/polym18040504

thermal treatment, filled with rosemary extract, f) no thermal treatment, g) 8 hours thermal treatment, h) 16 hours thermal treatment, i) 32 hours thermal treatment, and filled with spirulina biomass, j) no thermal treatment, k) 8 hours thermal treatment, l) 16 hours thermal treatment, m) 32 hours thermal treatment

https://doi.org/10.3390/polym18040504
